# A semi‐dominant NLR allele regulates growth and disease resistance in wheat

**DOI:** 10.1111/pbi.70244

**Published:** 2025-07-17

**Authors:** Shuiquan Tian, Tingting Du, Jianqing Niu, Shusong Zheng, Zhimeng Zhang, Hongwei Li, Qian‐Hua Shen, Hong‐Qing Ling, Yaoqi Si

**Affiliations:** ^1^ Institute of Genetics and Developmental Biology, Chinese Academy of Sciences Beijing China; ^2^ College of Advanced Agricultural Sciences, University of Chinese Academy of Sciences Beijing China; ^3^ Yazhouwan National Laboratory Sanya Hainan Province China

**Keywords:** Wheat, powdery mildew resistance, NLR, phenylalanine ammonia‐lyase, salicylic acid, lesion mimic

## Abstract

Wheat powdery mildew is a significant threat to wheat production, necessitating the development of disease‐resistant varieties as an economically viable and environmentally sustainable strategy. In this study, we investigated a semi‐dominant mutant, *necrosis leaf* (*necl*), which exhibits spontaneous necrotic lesions and enhanced resistance to powdery mildew. We identified that the *necl* phenotype is caused by a Lys‐to‐Glu gain‐of‐function mutation at position 421 in the coiled‐coil nucleotide‐binding leucine‐rich repeat (CC‐NLR) protein TaCNL, through the combination of map‐based cloning, transformation, and mutagenesis. Further analysis indicated that the TaCNL mutant enhanced resistance to powdery mildew likely through activation of the phenylalanine catabolic process and increased salicylic acid levels. Importantly, artificially modifying the amino acid at position 421 of TaCNL to an acidic residue induces immune necrosis, suggesting a potential strategy for engineering disease‐resistant proteins. These findings provide novel insights into the dual role of TaCNL in modulating growth and defence in wheat and offer a valuable genetic resource for developing durable resistance in wheat.

## Introduction

Plants are constantly challenged by a diverse array of microbial pathogens and insect herbivores, prompting the evolution of sophisticated defence mechanisms to counteract these threats (Dangl and Jones, 2001; Du *et al*., 2009; Wang *et al*., 2021). Central to these defences are resistance (R) proteins, which, upon activation, trigger a form of programmed cell death (PCD) known as the hypersensitive response at the site of infection, thereby limiting pathogen spread (Coll *et al*., 2014; Kourelis and van der Hoorn, 2018; Wan *et al*., 2019; Wu *et al*., 2017).

PCD in plants is a well‐characterized process activated by the recognition of pathogen‐derived effectors by nucleotide‐binding leucine‐rich repeat (NLR) proteins, which are the main types of R proteins (Manser *et al*., 2023; Salcedo *et al*., 2017; Scheel, 1998). NLR proteins are classified into two categories based on the secondary structure of their N‐terminus: those with a Toll‐interleukin 1 receptor domain (TIR‐NLR) and those with a coiled‐coil domain (CC‐NLR) (Jones and Dangl, 2006; Kasmi *et al*., 2017; Van Ooijen *et al*., 2008; Wang *et al*., 2021). Notably, dicotyledonous plants possess both CC‐NLR and TIR‐NLR proteins, while monocotyledonous species, such as wheat, harbour only CC‐NLR proteins (Pan *et al*., 2000; Tang *et al*., 2011).

Resistance proteins are thought to exist in an autoinhibited conformation, remaining inactive in the absence of pathogenic stimuli (Bi *et al*., 2021; Wang *et al*., 2021). Upon pathogen detection, NLR proteins recognize avirulence (Avr) effectors secreted by the pathogens, either directly or indirectly, initiating a cascade of defence responses (Dangl and Jones, 2001; Salcedo *et al*., 2017). These responses are characterized by the rapid generation of reactive oxygen species (ROS) in infected cells (Hammond‐Kosack and Jones, 1996; Zhang *et al*., 2023a; Zou *et al*., 2018). Concurrently, these defensive reactions are associated with the accumulation of salicylic acid (SA), which induces expression of a wide range of pathogenesis‐related (*PR*) genes and establishes systemic acquired resistance (SAR) (Durrant and Dong, 2004; Noutoshi *et al*., 2005).

Recent studies have increasingly revealed that mutations within NLR proteins can activate defence responses in the absence of pathogenic agents. For example, specific amino acid substitutions within CC‐NLR proteins NLS1‐1 and SCR8 in rice have been shown to result in the constitutive expression of defence responses, characterized by extensive cell death, elevated hydrogen peroxide levels, SA accumulation, heightened expression of *PR* genes, and enhanced resistance to *Xanthomonas oryzae* pv. *Oryzae* (Hu *et al*., 2021; Tang *et al*., 2011). In wheat, a mutation altering Arg to His at the 790th amino acid in a plasma membrane‐localized CC‐NLR protein RPM1 has been observed to induce exaggerated immune responses, leading to premature leaf senescence and up‐regulation of senescence‐ and autophagy‐associated genes (Zhang *et al*., 2023b). Additionally, a point mutation from Asp to Asn at the 441st amino acid in the CC‐NLR protein DES1 disrupts its interaction with replication protein A, resulting in aberrant mitotic cell division and ultimately causing plant mortality (Jia *et al*., 2021). Overexpression of NLR genes, such as tomato *Pto* or *Prf*, can also elicit pathogen‐independent resistance responses (Oldroyd and Staskawicz, 1998). Furthermore, the introduction of an NLR gene into a specific genetic background can induce pathogen‐independent resistance responses (Bomblies, 2007; Chae *et al*., 2014; Si *et al*., 2021). In the context of hybrid necrosis, the co‐presence of an NLR gene and its paired necrosis gene induces necrotic reactions, whereas individual NLR genes do not elicit an immune response (Chae *et al*., 2014; Yan *et al*., 2021). The *Ne2* gene, identified as a prototypical CC‐NLR protein, does not induce necrosis when overexpressed in isolation, but leads to leaf necrosis and whole plant necrosis in the presence of *Ne1*, accompanied by the up‐regulation of *PR* genes and accumulation of hydrogen peroxide (H_2_O_2_) (Si *et al*., 2021).

Among the approximately 20 genes identified as conferring resistance to wheat diseases, the majority encode NLR proteins (Jia *et al*., 2021). These resistance genes have predominantly been isolated through the exploration of genetic diversity among wheat cultivars. However, fewer NLR protein variants with constitutive activation have been identified in wheat. This study introduces the semi‐dominant wheat mutant *necl*, which exhibits tissue‐specific and age‐dependent spontaneous necrotic lesions along with defence‐related phenotypes. Using map‐based cloning techniques, we identified a single amino acid substitution within the NB domain of a CC‐NLR protein responsible for the *necl* mutant phenotype. Further investigation indicated that the increased SA levels in the *necl* mutant may bolster its resistance to wheat powdery mildew.

## Results

### Characterization of the *necl* mutant

**Figure 1:**
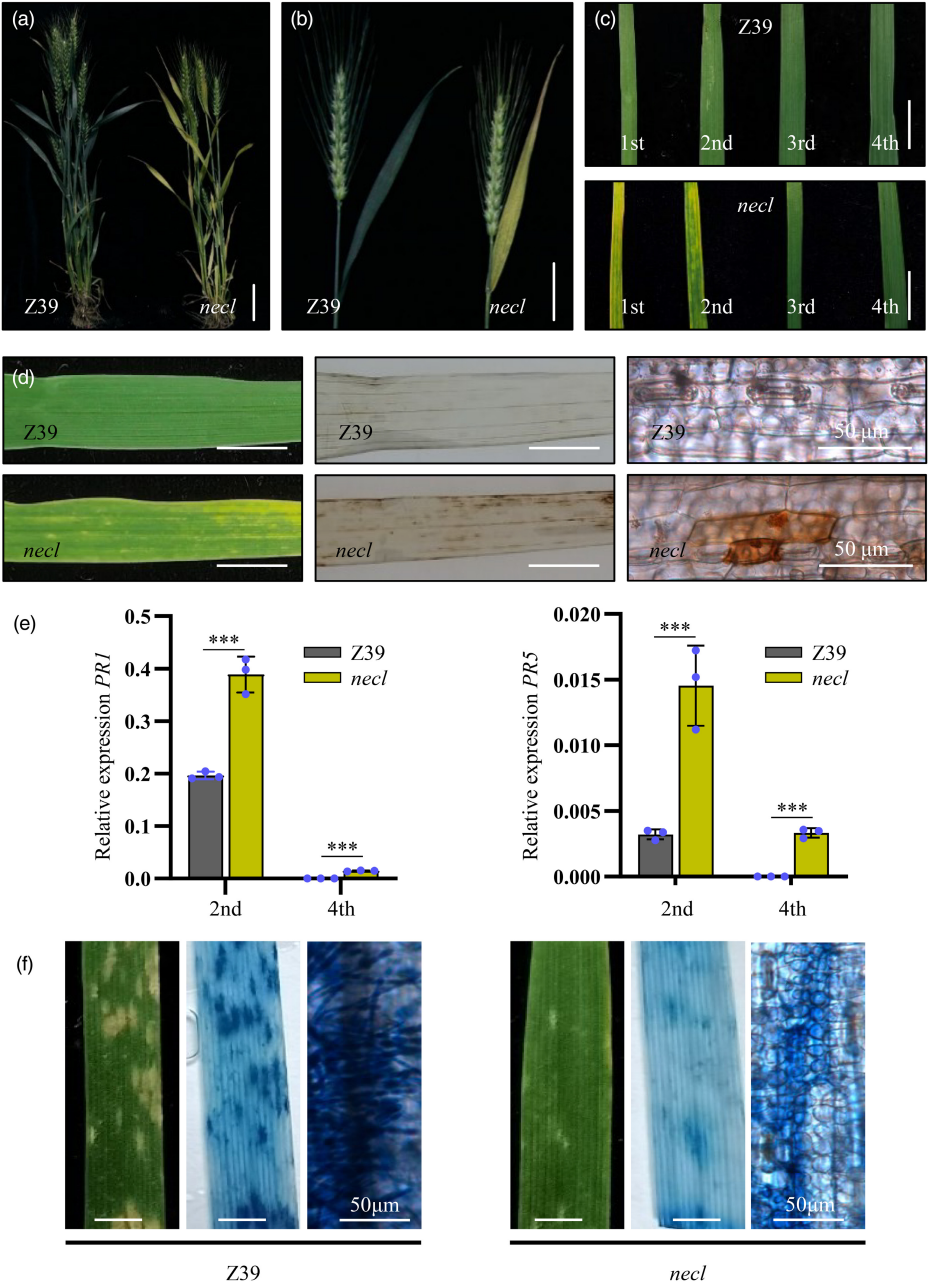
The *necl* mutant causes constitutive activation of defence responses in wheat. (a) Phenotypes of Z39 and the *necl* mutant at the flowering stage. Scale bar, 10 cm. (b) Spike and leaf morphology of the *necl* mutant. Scale bar, 5 cm. (c) Lesion severity correlates with leaf age. 1st, 2nd, 3rd and 4th represent the first, second, third, and fourth leaf, respectively, from the bottom at the tillering stage (30 days after sowing). Scale bar, 1 cm. (d) The accumulation of H_2_O_2_ in the leaves of Z39 (up panel) and the *necl* mutant (below panel) was observed by 3,3′‐diaminobenzidine (DAB) staining in the absence of pathogen infection. Scale bar, 1 cm. (e) Expression patterns of pathogenesis‐related marker genes *PR1* and *PR5* in the 2nd and 4th leaf of Z39 and the *necl* mutant (shown in c). 2nd and 4th represent the second and fourth leaf, respectively, from the bottom at the tillering stage (30 days after sowing). Error bars represent ± SD of the values of three biological repeats. Student's *t*‐test was used for statistical significance analysis. ***, *P* < 0.001. (f) The *necl* mutant displayed enhanced powdery mildew resistance. Two‐week‐old Z39 and *necl* mutant plants were inoculated with *Bgt* isolate E09, and representative leaves were photographed at 7 days post‐inoculation. Cell death was observed in wheat Z39 and *necl* mutant by trypan blue staining. Scale bar, 0.5 cm.

The *necrosis leaf* (*necl*) mutant was identified from a low‐energy nitrogen‐ion (N^+^) beam‐mutagenized population of the hexaploid wheat line Z39. The *necl* mutant exhibits necrotic lesions on leaves during the heading and flowering stages (Figure 1a), with no significant changes in spike or leaf morphology (Figure 1b). During the tillering stage, lesions typically initiate at the leaf tips and progress towards the base, with severity correlating with leaf age (Figure S1a). Basal leaves show more aggressive necrosis, while upper leaves exhibit milder lesions (Figure 1c). Additionally, plant height, tiller number, and thousand‐kernel weight were significantly reduced in the *necl* mutant compared to Z39, with no significant difference in spikelet number (Figure S1b). Lesion formation in the *necl* mutant is independent of environmental stress or pathogens. Excess H_2_O_2_ localized to lesions was observed in the *necl* mutant based on 3,3′‐diaminobenzidine (DAB) staining, with no such accumulation in Z39 plants (Figure 1d). These necrotic lesions resemble those seen during the hypersensitive response.

### Autoimmune activation in the *necl* mutant

Given the spontaneous hypersensitive response‐like lesions in the *necl* mutant, we hypothesized that defence responses were altered. To test this, we assessed the expression of pathogenesis‐related marker genes (*PR1* and *PR5*) in the *necl* mutant and Z39 plants. Compared to Z39, expression analysis revealed a significant up‐regulation of *PR1* and *PR5* both pre‐ and post‐lesion formation in the *necl* mutant (Figure 1e), indicating the activation of systemic acquired resistance. We further investigated whether the *necl* mutant exhibited enhanced disease resistance against virulent pathogens. Inoculation with *Blumeria graminis* f. sp. *tritici* (*Bgt*) isolate E09, which induces wheat powdery mildew, showed that the *necl* mutant displayed enhanced resistance compared to Z39 plants (Figures 1f and S1c).

### Map‐based cloning of the causal gene underlying the *necl* phenotype

To elucidate the genetic basis of the *necl* phenotype, we conducted crosses between *necl* and three wheat varieties: Z39, Jing 411 (J411), and Kenong 199 (KN199), yielding three distinct mapping populations. The F_1_ progeny of the *necl* × Z39 cross displayed intermediate necrosis (Figure S2), and the F_2:3_ populations exhibited a 1:2:1 Mendelian ratio of homozygous green, segregating, and homozygous necrotic individuals (*χ* ^2^ = 0.88 < *χ* ^2^_(1:2:1,0.05)_ = 5.99), confirming that a single semi‐dominant nuclear gene is responsible for the necrosis phenotype.

**Figure 2:**
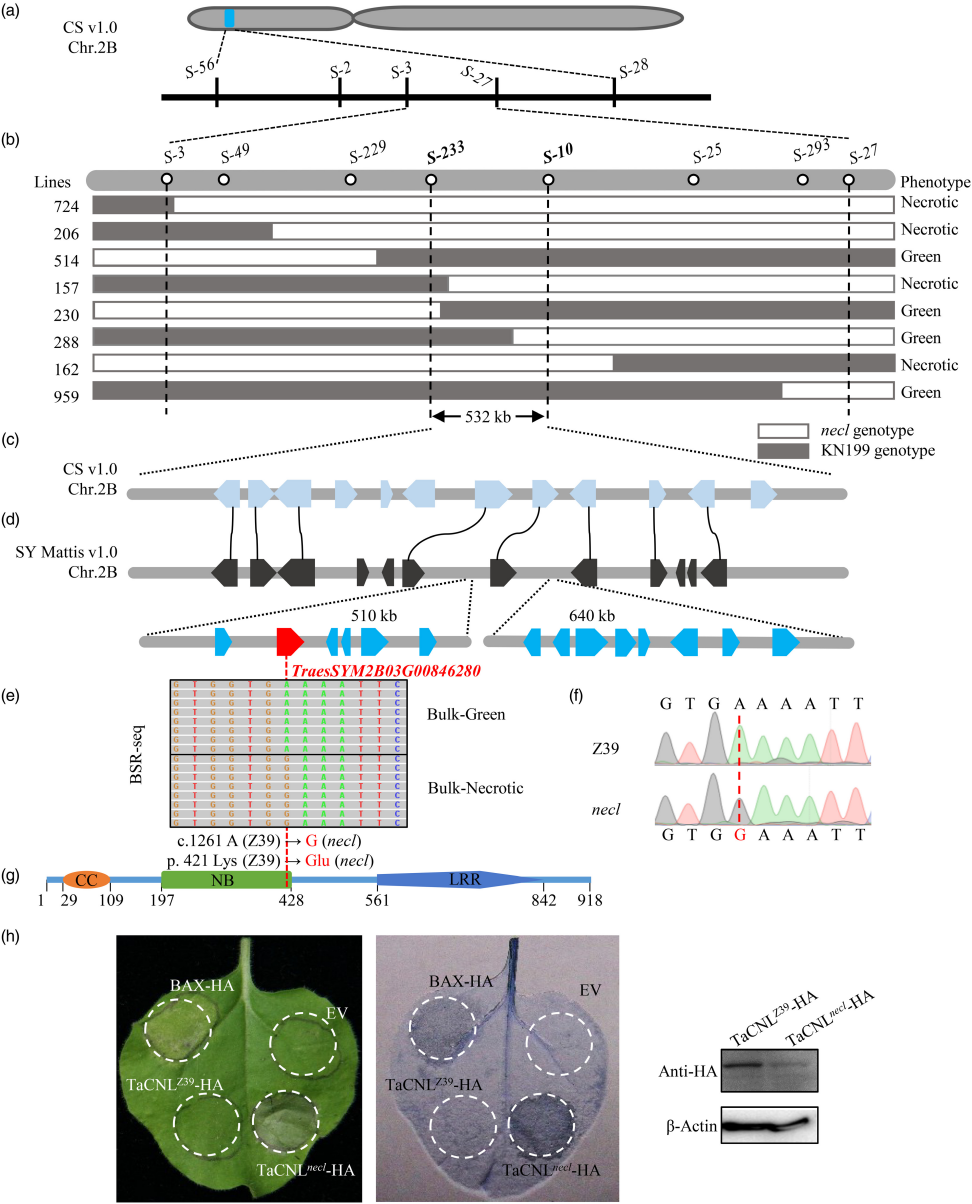
Map‐based cloning of the candidate gene for *necl* mutation. (a) The candidate gene was localized to chromosome 2B between markers *S‐3* and *S‐27* using 3620 F_2:3_ plants from the *necl* × J411 cross. (b) Schematic map of the key recombinants. (c) High‐confidence genes annotated within the mapping interval according to the Chinese Spring reference genome IWGSC Annotation v1.1. The 532‐kb genomic region between the markers *S‐233* and *S‐10* contains 12 predicted genes. (d) There are 26 high‐confidence genes within the candidate interval based on SY Mattis v1.0, which features two notable insertions within the target region compared to IWGSC RefSeq v1.0. A red pentagon indicates the *TaCNL* gene (*TraesSYM2B03G00846280*). (e) A single nucleotide polymorphism (SNP) (c. A1261G) in *TraesSYM2B03G00846280* was identified on SY Mattis v1.0 chromosome 2B by Bulked Segregant RNA‐seq (BSR‐seq) analysis between Bulk‐Green and Bulk‐Necrotic groups. Sequencing reads are visualized through IGV software. (f) Partial sequence of *TaCNL* from Z39 and the *necl* mutant. The mutant SNP (c. A1261G) is highlighted by the red dashed line. (g) Schematic representation of the TaCNL protein. CC, coiled‐coil; NB, nucleotide‐binding; LRR, leucine‐rich repeat. The mutation site is in the predicted NB domain. (h) TaCNL^*necl*^ induced cell death in *Nicotiana benthamiana* leaves. *p35S*::*TaCNL* ^*Z39*^ *‐HA* and *p35S*::*TaCNL* ^*necl*^ *‐HA* were transiently expressed in *N*. *benthamiana* leaves, respectively. The infiltrated leaves were photographed (left panel) and then stained with trypan blue (middle panel) to visualize cell death at 48 h post‐injection. Accumulation of proteins is shown in the right panel. The *BAX* gene was used as the positive control. EV, empty vector.

To identify the gene responsible for the *necl* phenotype, we genotyped 14 homozygous green and necrotic F_2:3_ individuals derived from the *necl* × J411 cross using the wheat 35 K SNP array. SNP‐phenotype associations were assessed using the Generalized Linear Model (GLM) in TASSEL Version 5.0, which identified significant SNPs associated with the *necl* phenotype within the 5.0–38.4 Mb region on chromosome 2B (Figure S3). Initial genetic linkage analysis with 3620 F_2:3_ plants from the *necl* × J411 cross mapped the mutated gene to a 3.5 Mb region between markers *S‐3* and *S‐27* (Figure 2a, Table S1). Further analysis with a larger F_2_ population (6865 plants) of the *necl* × KN199 cross localized the gene to a 532 kb interval between markers *S‐233* and *S‐10* (Figure 2b). There are 12 high‐confidence genes within this interval based on the IWGSC RefSeq v1.0 (Figure 2c, Table S2), but sequencing revealed no nucleotide variations between Z39 and the *necl* mutant (Table S1). Further investigation showed that some candidate genes in the *necl* mutant significantly differ from the IWGSC RefSeq v1.0 but align with the SY Mattis v1.0 reference sequence (Figure S4), indicating closer genomic affinity between the *necl* mutant and SY Mattis in the candidate region. Compared to IWGSC RefSeq v1.0, the SY Mattis v1.0 genome features two notable insertions within the target region, approximately 510 and 640 kb (Figure S5), encompassing six and eight high‐confidence genes (Figure 2d, Table S3), respectively. Bulked Segregant RNA sequencing (BSR‐Seq) analysis on 30 homozygous green (Bulk‐Green group) and 30 homozygous necrotic (Bulk‐Necrotic group) plants from the *necl* × J411 F_2:3_ population identified 14 candidate genes expressed in leaf tissue (Table S3). A unique SNP (c.1261 A > G) in *TraesSYM2B03G00846280*, causing a lysine to glutamic acid substitution at position 421 (p.421 Lys > Glu), was observed between the Bulk‐Green and Bulk‐Necrotic groups (Figure 2e). To validate this SNP, a marker was developed (Figure 2f). *TraesSYM2B03G00846280*, annotated as a CC‐NLR type protein of 918 amino acids in the SY Mattis v1.0 database, contains the mis‐sense mutation (Lys421Glu) within the nucleotide‐binding domain (Figure 2g).

Given that NLR protein mutations can induce defence responses and PCD in the absence of pathogens (Tang *et al*., 2011; Zhang *et al*., 2023b), we first employed a transient expression system in *Nicotiana benthamiana* leaves to assess the capacity of *TraesSYM2B03G00846280* to induce PCD. We designated the *TraesSYM2B03G00846280* gene from Z39 and the *necl* mutant as *TaCNL* ^*Z39*^ and *TaCNL* ^*necl*^, respectively. The transient expression results suggest that *TaCNL* ^*necl*^, but not *TaCNL* ^*Z39*^, can induce cell death in *N*. *benthamiana* leaf (Figure 2h), tentatively identifying *TaCNL* ^*necl*^ as the likely candidate gene responsible for the *necl* phenotype.

### Gain‐of‐function mis‐sense mutation in TaCNL causes auto‐activated hypersusceptibility necrosis

**Figure 3:**
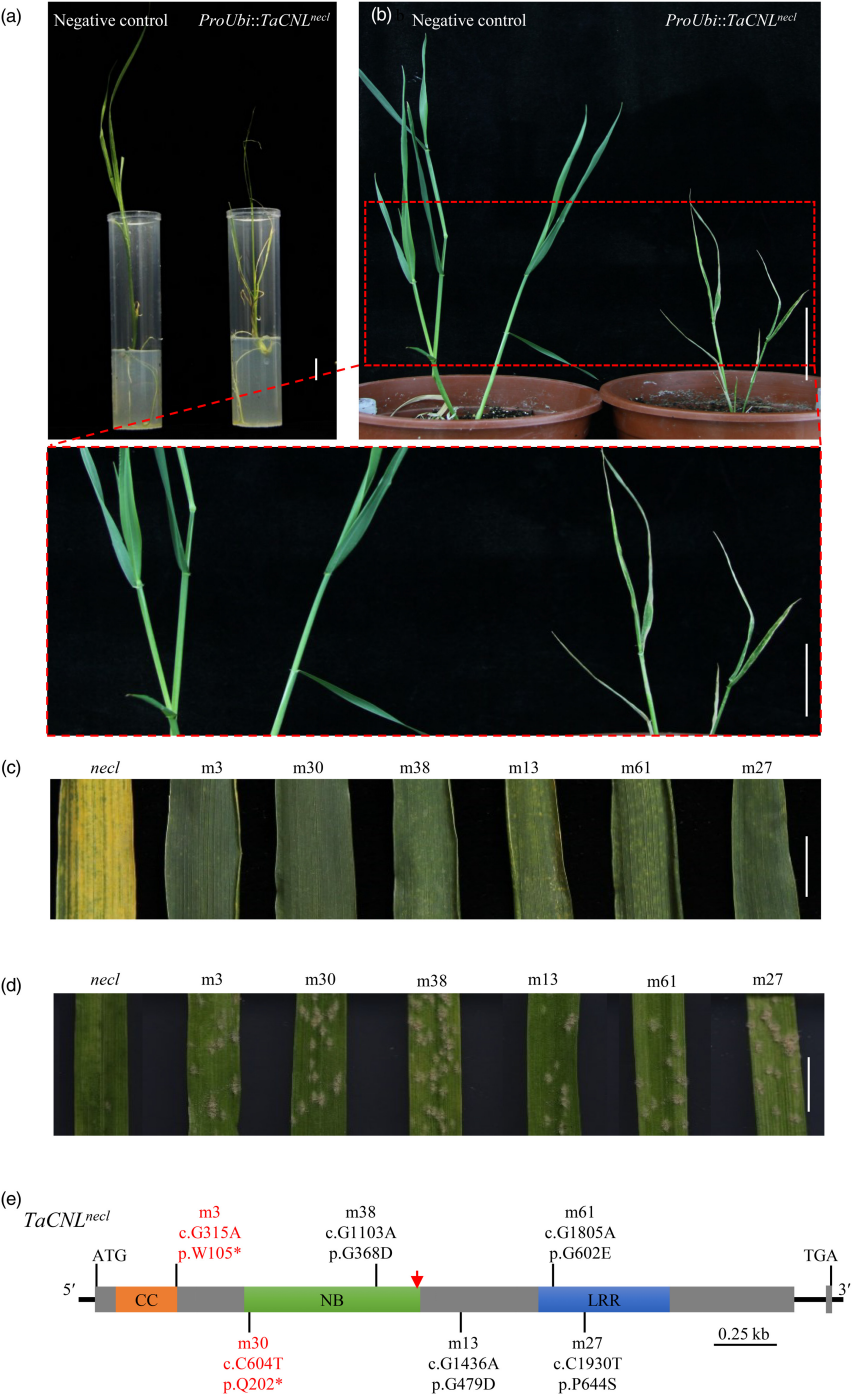
Function validation of *TaCNL* ^*necl*^ by transgenic assay and EMS mutants. (a, b) Transgenic T_0_ wheat plants expressing *TaCNL* ^*necl*^ in culture tubes (a, bar = 1 cm) and in the field 25 days after transplanting (b, bar = 9 cm; 1.7 × magnification, bar = 5.3 cm). The plant on the right is a positive transgenic (*proUbi*::*TaCNL* ^*necl*^), while the plant on the left is a nontransgenic plant (Negative control). (c) Phenotypes of the *necl* mutant and six non‐necrotic mutants derived from the *necl* background through ethyl methanesulfonate (EMS) mutagenesis, observed at the grain filling stage. Scale bar, 1 cm. (d) Non‐necrotic mutants showed reduced resistance to wheat powdery mildew. Two‐week‐old non‐necrotic mutants derived from the *necl* background were inoculated with *Bgt* isolate E09, and representative leaves were photographed at 7 d post‐inoculation. Scale bar, 5 mm. (e) Gene structure and EMS mutant analysis of *TaCNL* ^*necl*^. The positions of the EMS‐derived mutations are indicated by black lines. Grey boxes and black straight lines represent exons and introns, respectively. The coding sequence (c.) changes and their predicted effects on the amino acid (p.) are indicated. Mutation names in red indicate non‐sense mutations. CC, coiled‐coil; NB, nucleotide‐binding; LRR, leucine‐rich repeat. The mutation site (c. A1261G) in *TaCNL* ^*necl*^ is pointed out by a red arrow.

To confirm whether the point mutation (A1261G) identified in *TaCNL* corresponded to the *necl* phenotype, we conducted an overexpression assay and ethyl methanesulfonate (EMS) mutagenesis. For the overexpression assay, the full‐length coding sequence of *TaCNL* ^*necl*^ was introduced into the common wheat cultivar Fielder under the control of a ubiquitin promoter via *Agrobacterium*‐mediated transformation. The overexpression transgenic plants exhibited a lesion mimic phenotype and were severely weakened (Figure 3a), displaying dwarfism during early vegetative development and eventually dying after 25 days of growth in soil (Figure 3b), thus demonstrating the hyper‐susceptible necrosis effect of *TaCNL* ^*necl*^ in wheat.

To further evaluate the impact of TaCNL loss‐ or gain‐of‐function on the hypersensitive response in the *necl* mutant, seeds from the *necl* mutant were subjected to mutagenesis with 0.5% EMS. Field screening of approximately 5500 M_2_ individuals identified 6 M_2_ lines with a non‐necrotic phenotype (Figure 3c). Consistently, the six non‐necrotic mutants lost resistance to wheat powdery mildew (Figure 3d). Sequence analysis revealed distinct EMS‐induced single nucleotide transitions (G/C to A/T) in the coding sequence of *TaCNL* ^*necl*^ from all non‐necrotic mutants (Figure 3c–e). Three mutants (m38, m61 and m27) harboured mis‐sense mutations in conserved domains (NB and LRR), while one mutant (m13) had a mutation in the junctions of functional domains (Figure 3e). Two non‐necrotic mutants (m3 and m30) carried non‐sense mutations, leading to a premature stop codon at the amino acid positions 105 and 202, respectively (Figure 3e). Since these non‐sense mutations occurred upstream of the 421Glu site in TaCNL^*necl*^ (Figure 3e), they are classified as loss‐of‐function mutations of TaCNL^*necl*^ or TaCNL^Z39^. Therefore, the hypersusceptibility necrosis in the *necl* mutant is attributed to a gain‐of‐function effect of the TaCNL^*necl*^ mutation.

### Expression pattern and subcellular localization

**Figure 4:**
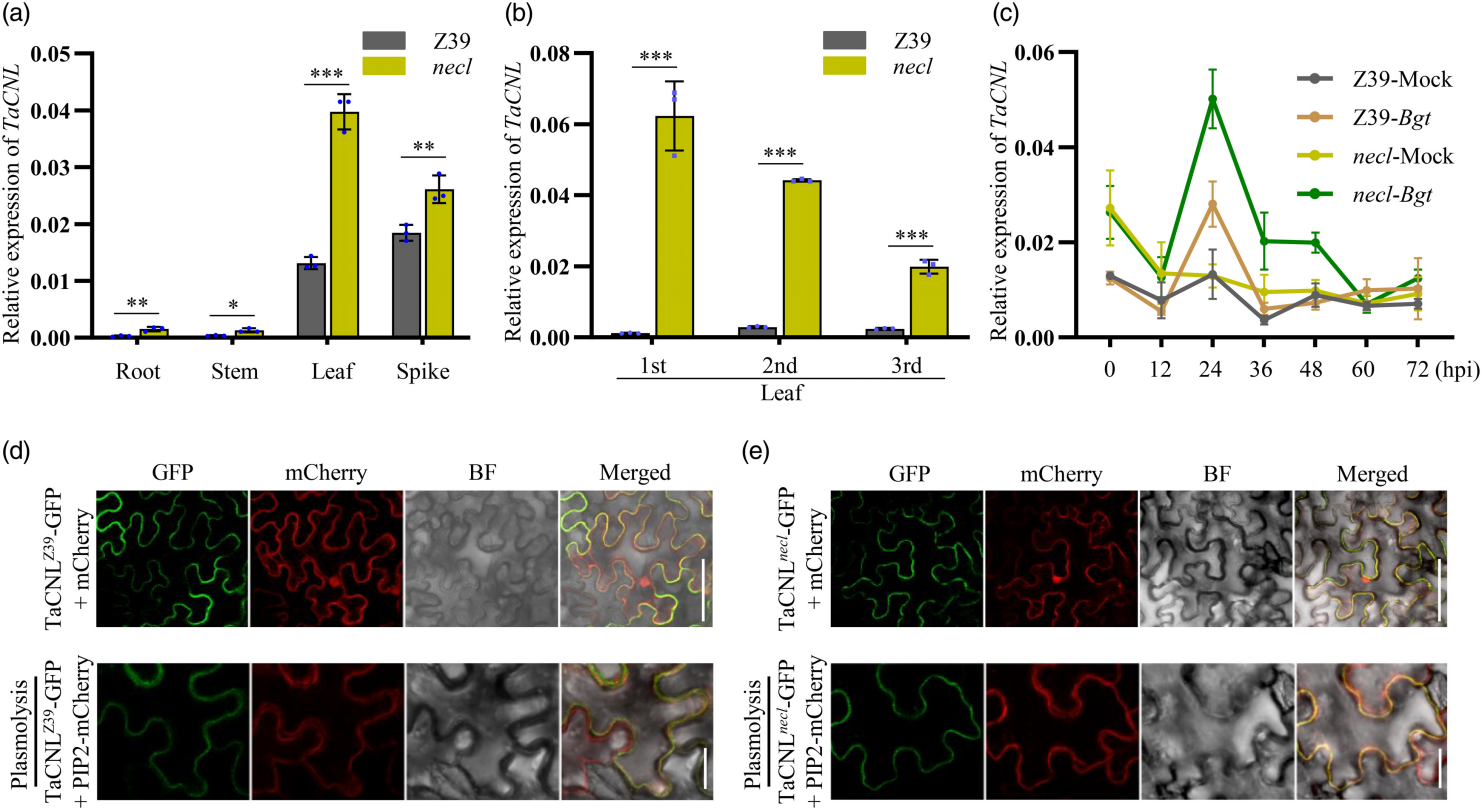
Expression pattern analyses and subcellular localization of *TaCNL*. (a) The expression pattern of *TaCNL* was examined in different tissues of Z39 and the *necl* mutant. Root samples were collected from plants grown for two weeks. Samples of stem, leaf and spike were collected at the flowering stage. (b) Expression profiles of *TaCNL* in the leaves of Z39 and the *necl* mutant at different developmental stages. Leaf‐1st, 2nd and 3rd represent the first, second and third leaf, respectively, from the bottom at the tillering stage (30 days after sowing). (c) *TaCNL* expression in Z39 and the *necl* mutant plants at different times (hours) post‐inoculation (hpi) with *Bgt* isolate E09, measured by quantitative reverse transcription (qRT)‐PCR. *TaActin* gene was used as an endogenous control. Data in a– c are the mean ± SD of values obtained from three independent biological samples. Student's *t*‐test was used for statistical significance analysis. *, ** and *** indicate a statistically significant difference at *P* < 0.05, *P* < 0.01 and *P* < 0.001 levels, respectively. (d, e) The Lys421Glu amino acid substitution in TaCNL does not impact its subcellular localization. In *N*. *benthamiana* leaf epidermal cells, the TaCNL^Z39^‐GFP (d) and TaCNL^*necl*^‐GFP (e) fusion proteins exhibit partial colocalization with mCherry in the cytoplasm and with PIP2‐mCherry (a plasma membrane marker) in the plasma membrane, respectively. Plasmolysis was achieved by incubating cells in 20% Manitol. Scale bar, 5 μm.

To characterize the spatial expression profile of *TaCNL*, we performed quantitative reverse transcription (qRT)‐PCR analysis. The results revealed widespread expression in various tissues of wheat, including roots, stems, flag leaves and spikes, with the highest expression detected in the flag leaves (Figure 4a). Interestingly, lower leaves exhibited significantly higher levels of *TaCNL* ^*necl*^ expression compared to adjacent upper leaves, showing a positive correlation with the severity of necrosis (Figures 1c and 4b). Moreover, mutation in *TaCNL* resulted in an up‐regulation of its expression levels (Figure 4a,b). Since the *necl* mutant demonstrated enhanced resistance to powdery mildew, we conducted inoculation experiments using the *Bgt* isolate E09. The results showed a significant increase in *TaCNL* ^*necl*^ expression in the mutant at 24 h post‐inoculation, indicating the responsiveness of *TaCNL* ^*necl*^ to the pathogen (Figure 4c).

*TaCNL* encodes a CC‐NLR type protein with 918 amino acids, and the mis‐sense Lys421Glu mutation in the *necl* mutant is located in the NB domain (Figure 2g). Additionally, the Lys421Glu substitution in TaCNL is predicted to cause significant expansion of the cavity volume in the protein (Figure S6), as suggested by analysis using the AlphaFold Protein Structure Database (https://alphafold.ebi.ac.uk/) and Missense3D (http://missense3d.bc.ic.ac.uk/missense3d/). To investigate whether the Lys421Glu substitution affects the subcellular localization of TaCNL, we analysed the subcellular localization of TaCNL^Z39^ and TaCNL^*necl*^ by separately co‐expressing *p35S*::*TaCNL* ^*Z39*^‐*green fluorescent protein* (*GFP*) or *p35S*::*TaCNL* ^*necl*^‐*GFP* with red fluorescent protein (*p35S*::*mCherry* and *p35S*::*PIP2‐mCherry*) in the epidermal cells of *N*. *benthamiana* leaves. Fluorescence analysis revealed that the fluorescence from the TaCNL^Z39^‐GFP and TaCNL^*necl*^‐GFP fusion proteins exhibits partial colocalization with mCherry in the cytoplasm and with PIP2‐mCherry (a plasma membrane marker) in the plasma membrane (Figure 4d,e). This observation indicates that both TaCNL^*Z39*^ and TaCNL^*necl*^ are predominantly localized in the cytoplasm and plasma membrane and that the Lys421Glu substitution does not affect the subcellular localization of TaCNL.

### The TaCNL mutant enhanced resistance to powdery mildew likely by activating the phenylalanine catabolic process and increasing salicylic acid levels

**Figure 5:**
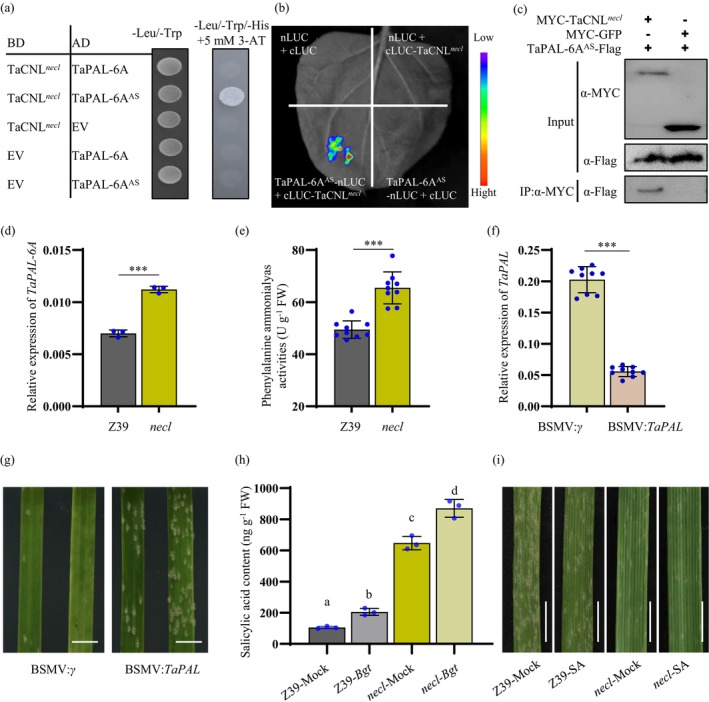
TaCNL mutant enhances powdery mildew resistance in wheat through phenylalanine catabolic process and SA signalling pathway. (a) Yeast two‐hybrid assays showing the interactions between TaCNL^*necl*^ and the active site region (TaPAL‐6A^AS^, residues 107–400) of TaPAL‐6A. (b) The interactions of TaCNL^*necl*^ and TaPAL‐6A^AS^ detected by split luciferase complementation assays. (c) Co‐immunoprecipitation assay demonstrating the interaction between TaCNL^*necl*^ and TaPAL‐6A^AS^ in *N*. *benthamiana* leaves. IP, immunoprecipitation. (d, e) The TaPAL‐6A expression levels (d, *n* = 3) and phenylalanine ammonia‐lyase activities (e, *n* = 9) analysis of the leaves from Z39 and the *necl* mutant at the tillering stage (30 days after sowing). (f) Relative expression levels of *TaPAL* in the *necl* mutant treated with BSMV:*γ* (control) and BSMV:*TaPAL*, respectively. Three leaves used per biological replicate (*n* = 9). (g) The BSMV:*γ* and BSMV:*TaPAL* plants, derived from the *necl* mutant background, were inoculated with the *Bgt* isolate E09. Representative leaves were harvested and photographed at 7 days post‐inoculation. Scale bar, 5 mm. (h) Salicylic acid (SA) contents in leaves of two‐week‐old Z39 and the *necl* mutants. Z39‐*Bgt* and *necl*‐*Bgt* denote SA measurements taken 24 h post‐inoculation with *Bgt* isolate E09. Results represent the means ± SD (*n* = 3), and different letters indicate significant differences between groups, as determined by one‐way ANOVA, Tukey's post hoc test (*p* < 0.05). (i) Exogenous application of SA enhances resistance to powdery mildew in wheat. Two‐week‐old wheat seedlings were sprayed with 200 mM SA solution or an equivalent volume of H_2_O as a control and inoculated with *Bgt* isolate E09 after 24 h. Representative leaves were harvested and photographed 7 days post‐inoculation. Scale bar, 5 mm. Results in d‐f represent the means ± SD, and student's *t*‐test was used for statistical significance analysis. *** indicates a statistically significant difference at *P* < 0.001 level.

To investigate the potential mechanisms by which TaCNL^*necl*^ regulates wheat immunity, we screened a yeast two‐hybrid (Y2H) library constructed from the wheat line Z39 using the full‐length TaCNL^*necl*^ to identify candidate interacting proteins. Among the identified interactors, phenylalanine ammonia‐lyase (TaPAL‐6A, TraesCS6A02G222800) was found to specifically interact with TaCNL^*necl*^. Further 1:1 Y2H assays confirmed that TaCNL^*necl*^ exclusively interacts with the active site region (TaPAL‐6A^AS^, residues 107–400) of TaPAL‐6A (Figure 5a). The interaction between TaCNL^*necl*^ and TaPAL‐6A^AS^ was further validated in vivo using luciferase complementation imaging (LCI) and co‐immunoprecipitation (Co‐IP) assays in *N*. *benthamiana* epidermal cells (Figure 5b,c). Similar interactions were observed between TaCNL^Z39^ and TaPAL‐6A^AS^ (Figure S7). Together, these results demonstrate that TaCNL physically interacts with TaPAL‐6A.

PAL is a key enzyme in the phenylpropanoid metabolism pathway, involved in the synthesis of important secondary metabolites such as lignin, isoflavonoid phytoalexins and flavonoid pigments, which are crucial for plant defence against pathogens (Xu *et al*., 2024). To investigate the role of *TaPAL‐6A* in powdery mildew resistance in the *necl* mutant, we analysed RNA‐seq data from Bulk‐Green and Bulk‐Necrotic plants, revealing 6821 differentially expressed genes (DEGs) between the two groups (Table S4). Gene ontology (GO) analysis showed significant enrichment of defence response‐related genes (Figure S8, Table S5) and up‐regulation of the phenylalanine catabolic process (GO:0006559) in the Bulk‐Necrotic group (Table S6). Correspondingly, the expression and enzymatic activity of TaPAL‐6A were significantly higher in the *necl* mutant than in Z39 plants (Figure 5d,e). Additionally, downstream pathway genes associated with PAL in the *necl* mutant, including those involved in phenylpropanoid biosynthesis, flavonoid biosynthesis, phenylalanine metabolism and isoflavonoid biosynthesis, showed significant differences compared to those in Z39 (Table S7). These results suggest that the TaCNL mutation activates the phenylalanine catabolic process, either directly or indirectly. To confirm the role of *TaPAL‐6A* in powdery mildew resistance, we conducted virus‐induced gene silencing (VIGS) targeting the active site of TaPAL in the *necl* mutant. *TaPAL* transcript levels were significantly reduced in BSMV:*TaPAL* plants compared to BSMV:*γ* controls (Figure 5f). Consequently, *TaPAL*‐silenced plants exhibited compromised resistance to the *Bgt* isolate E09, with increased fungal biomass (Figure 5g). These findings indicate that *TaPAL‐6A* is essential for powdery mildew resistance in the *necl* mutant.

Notably, we observed significant enrichment of DEGs in the SA biosynthetic process (GO:0009697), SA‐mediated signalling pathway (GO:0009862) and SA metabolic process (GO:0009696) (Figure S8 and Table S5). Given that SA is a crucial hormone in plant immune responses and resistance to biotic stresses (Cass *et al*., 2015; Liu *et al*., 2023), we hypothesized that the TaCNL mutation might influence SA metabolism in the *necl* mutant. Quantification of SA levels in leaves showed significantly higher SA accumulation in the *necl* mutant than in Z39 (Figure 5h). Moreover, inoculation with *Bgt* isolate E09 induced SA accumulation in the leaves of both the *necl* mutant and Z39 plants (Figure 5h). These findings suggest that elevated SA levels may contribute to the enhanced powdery mildew resistance in the *necl* mutant. To test this hypothesis, we applied 200 mM SA to the *necl* mutant and Z39 seedlings 24 h prior to inoculation. The results revealed that exogenous application of SA significantly enhanced powdery mildew resistance in Z39 (Figure 5i). Thus, the enhanced resistance in the *necl* mutant is likely, at least partly, attributable to increased endogenous SA levels.

### Distribution and variation of *TaCNL*

To investigate the genetic diversity of *TaCNL*, we screened 413 wheat accessions from around the world using markers *S‐326* and *S‐48*. The results revealed that 165 of these accessions contained *TaCNL* (Table S8). Subsequently, we randomly selected 15 wheat accessions for the amplification and sequencing of *TaCNL*, identifying two distinct alleles of *TaCNL* (Table S9). One allele was identical to the *TaCNL* sequence found in Z39, while the other exhibited a single nucleotide polymorphism (SNP) at position 1263 in the coding region of *TaCNL* (c.1263A > C), leading to the substitution of lysine with asparagine (p.421 Lys > Asn) (Figure S9a), hereafter referred to as *TaCNL‐b*. Interestingly, both *TaCNL‐b* (Lys421Asn) and *TaCNL* ^*necl*^ (Lys421Glu) exhibited amino acid substitutions at position 421, compared to *TaCNL* ^*Z39*^. This prompted us to investigate whether *TaCNL‐b* could enhance disease resistance. To explore this, total of six wheat samples carrying the *TaCNL‐b* allele were screened and inoculated with the *Bgt* isolate E09. However, similar to Z39, powdery mildew resistance in these six wheat samples was not significantly improved (Figure S9b). Consistently, transient expression of the HA‐tagged TaCNL‐b fusion protein (TaCNL‐b‐HA) did not induce cell death in *N*. *benthamiana* leaves (Figure S9c).

### Effects of artificial mutant TaCNL

**Figure 6:**
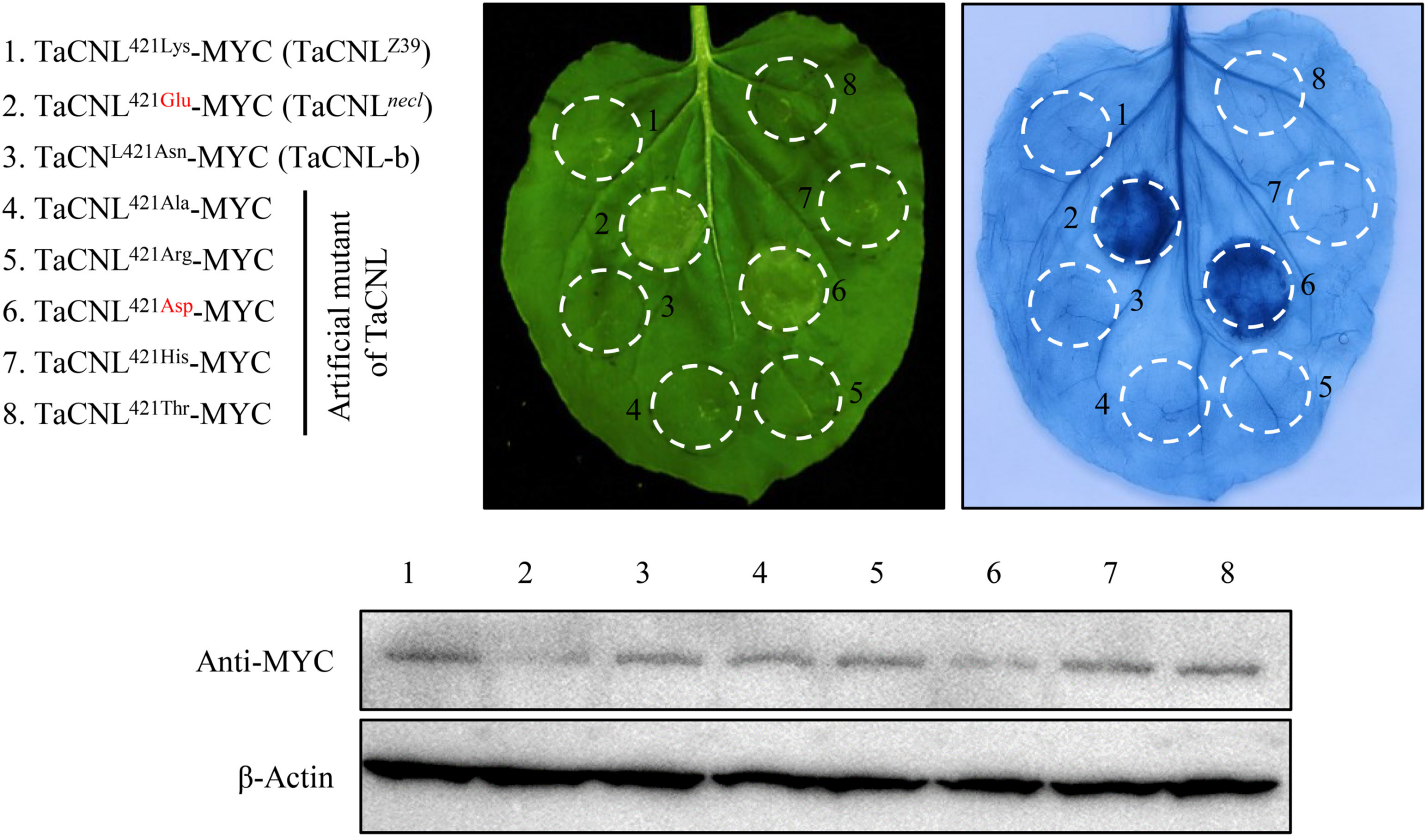
Effects of artificial mutant TaCNL. Multiple artificial TaCNL mutants were created by substituting 421Lys with basic (Arg and His), polar (Thr), nonpolar (Ala), and acidic (Asp) amino acids. These mutants were transiently expressed in *N*. *benthamiana* leaves, respectively. The infiltrated leaves were photographed (left panel) and then stained with trypan blue (right panel) to visualise cell death at 48 h post‐injection. Protein accumulation is shown in the bottom panel.

The 20 amino acids essential for life can be categorized into four groups: acidic, basic, polar and nonpolar. Further analysis revealed that Lys, Glu and Asn are classified as basic, acidic and polar amino acids, respectively. Therefore, the substitution of 421Lys (basic) in TaCNL^*necl*^ with Glu (acidic) may have a stronger impact than the substitution with Asn (polar). To test this hypothesis, we created multiple artificial mutants of TaCNL by substituting 421Lys with various amino acids, including basic (Arg and His), polar (Thr), nonpolar (Ala) and acidic (Asp) amino acids. When constructs expressing these artificial mutants were infiltrated into *N*. *benthamiana* leaves, cell death was observed only at the injection site for the Lys421Asp substitution at 48 h post‐injection (Figure 6). These results suggest that the substitution of 421Lys of the TaCNL protein with an acidic amino acid induces a hypersensitive response.

## Discussion

### Choosing a suitable reference genome for the research

The selection of an appropriate reference genome is a critical step in gene cloning research, particularly for complex polyploid species such as wheat (*Triticum aestivum* L.), which is an allohexaploid (2n = 6x = 42, AABBDD) comprising three subgenomes (A, B, D) that have evolved through polyploidy and subsequent integration (Appels *et al*., 2018). These subgenomes exhibit over 80% sequence similarity, and the wheat genome is characterized by a high proportion of repetitive sequences, accounting for more than 85% of its genomic content (Appels *et al*., 2018). This complexity poses significant challenges for sequencing and assembling a reference genome.

The landscape of wheat reference genomes has been significantly advanced with the release of the hexaploid Chinese Spring reference genome by the International Wheat Genome Sequencing Consortium (IWGSC) in 2018 (Appels *et al*., 2018), followed by the 10+ Genome Reference Project in 2020 (Walkowiak *et al*., 2020), and the release of the pan‐genome of 17 wheat cultivars from China in 2024 (Jiao *et al*., 2025). Initially, utilising the IWGSC RefSeq v1.0, we successfully completed early gene mapping. However, upon narrowing the target region to a 532 kb segment, the target gene remained elusive. The subsequent availability of the Zang1817 and 10+ genome references enabled us to conduct a comparative mapping of the candidate region across multiple genomes (Guo *et al*., 2020; Walkowiak *et al*., 2020), thereby uncovering potential structural variations within this region (Figure S10). Further sequencing and multiple alignment of the candidate genes led to the conclusion that the SY mattis v1.0 genome was more suitable as the reference genome, at least for the candidate region associated with the *necl* mutant. In the SY mattis v1.0 genome, in contrast to the IWGSC RefSeq v1.0, we identified two large insertion fragments within the target region (Figures 2c,d and S5). Phylogenetic analysis suggests that these insertions may also be present in the ancestral B subgenome (Figure S11). Consequently, we were able to rapidly pinpoint the candidate genes based on the SY mattis v1.0 genome.

This finding underscores the unique genomic attributes of each wheat material and highlights the limitations of relying on a single reference genome to fully represent the material under study. It is evident that a comparative approach, involving the analysis of multiple reference genomes, is indispensable for selecting the most appropriate reference genome. This not only circumvents potential missteps in the research process but also emphasizes the pivotal role of comparative genomics and pan‐genomics in advancing our understanding of complex polyploid genomes such as that of wheat.

### The *necl* phenotype may be associated with the burst of reactive oxygen species

It has been reported that the NB domain functions as a molecular switch in NLR proteins, and mutations within this domain can lead to auto‐activation of the protein. For example, a mutation in the NB domain of MLA10 in barley results in auto‐activation, which, when transiently expressed in *N*. *benthamiana* leaves, causes cell death (Bai *et al*., 2012). In the present study, the mutation in the *necl* mutant occurs in the NB domain of TaCNL. Expression of TaCNL^*necl*^ in both *N*. *benthamiana* and wheat induced cell death, confirming the occurrence of auto‐activation. DAB staining of the *necl* mutant leaves without pathogen inoculation revealed brown spots (Figure 1d), indicating the accumulation of H_2_O_2_, which corresponds to the plant's stress response associated with the activation of the ETI (Effector‐Triggered Immunity) pathway and the subsequent burst of reactive oxygen species.

It has been reported that NLR‐mediated hypersensitive responses depend on the accumulation of reactive oxygen species produced by the chloroplasts (Littlejohn *et al*., 2021). Chloroplasts possess a sophisticated antioxidant and self‐protection system that can mitigate oxidative stress caused by external stressors. The reactive oxygen species generated in the chloroplasts are crucial for triggering mechanisms that rapidly degrade damaged chloroplasts, thereby protecting the cell from the harmful effects of toxic by‐products in metabolism (Domínguez and Cejudo, 2021). Here, the RNA‐seq results revealed that, in addition to genes related to plant defence response being most enriched among the up‐regulated differentially expressed genes, genes associated with photosynthesis were down‐regulated in the Bulk‐Necrotic group (Figures S8 and S12; Tables S4 and S5). Furthermore, we observed that the chloroplasts in the *necl* mutant displayed abnormal ultrastructure (Figure S13). Although the overall outline was discernible, the internal structure appeared dim and indistinct, with irregular thylakoids that could not be clearly identified. Additionally, the starch granules were observed to be larger and more numerous in the *necl* mutant, indicating a deviant physiological state.

Based on these findings, it is inferred that the mutation in TaCNL^*necl*^ leads to constitutive auto‐activation in the *necl* mutant, initiating the wheat ETI response and promoting reactive oxygen species accumulation in the plant, particularly in the chloroplasts. The accumulation of reactive oxygen species in the chloroplasts triggers oxidative stress, activating mechanisms that lead to chloroplast degradation, resulting in abnormal chloroplast structures, chlorophyll degradation and ultimately leaf yellowing. This suggests that the *necl* phenotype is a consequence of the interplay between the auto‐activation of the NLR protein and the subsequent oxidative stress response in the chloroplasts. Further studies are needed to elucidate the specific molecular mechanisms underlying these processes and to explore potential strategies for modulating the reactive oxygen species response in plants.

### The potential biological significance of the TaCNL‐TaPAL pathway

Although the interaction between TaCNL and TaPAL‐6A may initially seem counterintuitive, similar interactions between resistance proteins and enzymes have been documented. For example, in rice, the small GTPase OsRac1 interacts with the NB‐ARC domain of the blast resistance protein Pit, playing a critical role in Pit‐mediated immune responses (Kawano *et al*., 2010). Additionally, in barley, the RING‐type E3 ligase MIR1 interacts with the N‐terminus of the CC‐NB‐LRR protein mildew A (MLA), mediating MLA degradation (Wang *et al*., 2016). Likewise, maize metacaspases modulate the defence response mediated by the NLR protein Rp1‐D21, likely through affecting its subcellular localization (Luan *et al*., 2021). In this study, we discovered that the significant increase in *TaPAL‐6A* expression observed in the *necl* mutant suggests that TaCNL^*necl*^ auto‐activation may indirectly activate *TaPAL‐6A* transcription through a signalling cascade. This could involve the activation of certain transcription factors or changes in their localization, thereby promoting the transcription of *TaPAL‐6A*. Similar cases have been reported; for example, the N‐terminal CC signalling domain of barley MLA interacts with two antagonistically acting transcription factors, MYB6 and WRKY1, to initiate disease resistance signalling (Chang *et al*., 2013). Likewise, the activated NLR protein PigmR promotes the accumulation of the RNA‐recognition motif‐containing transcription factor PIBP1 in the nucleus, where PIBP1 directly binds to the *OsPAL1* promoter and activates its transcription (Wang *et al*., 2019). Regrettably, we failed to identify similar transcription factors using the Y2H method. Future experiments employing immunoprecipitation‐mass spectrometry (IP‐MS) technology could potentially enable large‐scale identification of proteins that interact with TaCNL^*necl*^.

Moreover, we also found that there were interactions between TaPAL‐6A^AS^ and both kinds of TaCNL, while the specific activity of PAL in the *necl* leaves was significantly higher than that in Z39. Although this difference could be due to variations in PAL content, we cannot rule out the possibility of increased enzyme activity. Therefore, one plausible hypothesis is that TaCNL may act as an allosteric regulator of TaPAL‐6A. TaCNL^Z39^ binds to TaPAL‐6A in a dynamic equilibrium. However, either intrinsic structural rearrangements within TaCNL^*necl*^ or its interaction with a discrete binding site on TaPAL‐6A may induce conformational changes in TaPAL‐6A, thereby modulating the enzyme's catalytic efficiency. The enhancing overall enzyme activity promotes the biosynthesis of defence‐related compounds. Future experiments using in vitro enzyme activity assays, in which PAL protein levels are quantified and TaCNL protein or substrate of PAL is added, will help investigate the potential biological significance of their interaction.

The dual regulation of TaPAL‐6A at both the protein and transcriptional levels likely reflects the fine‐tuning required for plant defence responses. In the face of pathogen attack, plants need to rapidly and efficiently activate defence mechanisms. Direct protein‐level regulation allows for rapid modulation of TaPAL‐6A activity, while transcriptional regulation increases *TaPAL‐6A* expression, sustaining the defence response over a longer timescale.

### Potential applications in wheat breeding of *TaCNL* ^*necl*^

Wheat powdery mildew is a globally prevalent disease, and breeding resistant varieties is an economically effective strategy to control this disease. The main resistance genes used in major wheat varieties across different wheat‐growing regions in China include *Pm2*, *Pm4*, *Pm5*, *Pm8* and *Pm21* (Cao *et al*., 2015). However, in the ongoing arms race between pathogens and resistance genes, the evolved pathogens have gradually overcome the resistance provided by *Pm8* (Kunz *et al*., 2023), and other resistance loci are also at risk of being breached. The discovery and cloning of *TaCNL* ^*necl*^ will enrich the wheat powdery mildew resistance gene pool in China, although its practical application in production still requires further research and development.

While improving plant resistance to diseases, negative effects on other agronomic traits are often observed. For example, the *mlo* mutant in wheat confers durable and broad‐spectrum resistance to powdery mildew but is accompanied by growth disorders and yield loss (Acevedo‐Garcia *et al*., 2017). A new mutation type, *Tamlo‐32*, obtained through gene editing, maintains crop growth and yield while providing strong resistance to powdery mildew (Li *et al*., 2022). In this study, although the mutated gene, *TaCNL* ^*necl*^, enhanced wheat resistance to powdery mildew, it also caused leaf necrosis, ultimately leading to a reduction in important agronomic traits such as reduced tiller number and 1000‐kernel weight.

Here, we initially performed an artificial amino acid substitution mutation at position 421 of TaCNL, replacing Lys with Asp, and found that this substitution could also induce necrosis in *N*. *benthamiana*. This suggests that further research could be conducted to predict key functional amino acids in the TaCNL protein using artificial intelligence and to redesign a TaCNL protein that confers resistance to wheat powdery mildew without inducing immune necrosis. This approach has been successfully applied to rice, such as genome editing of rice CDP‐DAG synthase conferring resistance to multiple pathogens (Sha *et al*., 2023). Additionally, EMS mutagenesis could be applied again to the *necl* mutant to break the linkage between disease resistance and negative side effects, potentially screening for plants that are both resistant to disease and developmentally normal.

## Materials and methods

### Plant materials and phenotypic assessments

The *necrosis leaf*, *necl*, mutant was derived from a hexaploid wheat accession Z39 through mutagenesis induced by N^+^ ion beam irradiation. To elucidate the genetic basis of the *necl* phenotype, we conducted crosses between *necl* and three wheat varieties, including Z39, J411 and KN199, yielding three distinct mapping populations. Phenotypic assessments were conducted at the heading stage, with segregation populations categorized into two groups: plants exhibiting normal growth and those displaying necrotic leaves characteristic of the *necl* mutant. The phenotypes of critical recombinant plants were verified in their self‐pollinated progeny.

### Phenotypic response to powdery mildew

All seedlings to be inoculated were cultivated in 8 × 8 × 8 cm pots under aseptic conditions. Inoculation with *Bgt* E09 was performed at the two‐leaf stage of the tested plants under controlled greenhouse conditions maintained at 18–24 °C, with a 16 h light/8 h dark photoperiod and approximately 65% relative humidity. Infection types on the primary leaf of each plant were assessed at 7–10 days post‐inoculation (dpi). For experiments involving exogenous SA application, plants were sprayed with 200 mM SA (Solarbio, China) solution 24 h prior to inoculation. Control plants were sprayed with an equivalent volume of distilled water.

### Histochemical staining

DAB staining was performed according to a well‐established protocol with minor modifications (Xiao *et al*., 2003). Fresh leaves from both *necl* mutant and Z39 plants were immersed in a DAB solution (1 mg/mL DAB, 10 mM Na_2_HPO_4_, pH 5.8) in darkness for 12 h at ambient temperature. Subsequently, the leaves were decolorized in 95% ethanol to remove chlorophyll. Hyphae and leaf cell death in wheat were observed at 7 days post‐inoculation by trypan blue staining, as described previously (Koch and Slusarenko, 1990). To visualize cell death on *N*. *benthamiana* leaves, the infiltrated leaves were stained with trypan blue solution, followed by boiling for 10 min and overnight decolorization in chloral hydrate, as described before (Yuan *et al*., 2021). Each experimental condition was replicated three times.

### Map‐based cloning

Genomic DNA was extracted from young leaf tissues following a standardized procedure (Chatterjee *et al*., 2002). For the map‐based identification of the mutated gene, two parental lines and 14 homozygous green and necrotic F_2:3_ individuals from the J411 × *necl* cross were subjected to genotyping using the Breeders' 35 K Axiom® array (Affymetrix, #550524). An association analysis linking polymorphic SNPs to the necrosis phenotype was executed using the Generalized Linear Model function embedded in TASSEL Version 5.0. Alternatively, the threshold *P*‐value for selecting associated SNPs, based on the Bonferroni adjustment for independent SNPs, was set at 5 × 10^−5^. In the fine mapping phase, markers were designed, detected and utilized, following previously described methodologies (Si *et al*., 2022). Genome coordinates presented in this study are referenced to the IWGSC RefSeq v1.0 (Appels *et al*., 2018) and SY Mattis v1.0 (Walkowiak *et al*., 2020) assembly, and the gene annotations are based on the updated IWGSC Annotation v1.1 and SY Mattis Annotation PGSBv2.1. The primers used for map‐based cloning are detailed in Table S1.

### Gene expression analysis

Total RNA was isolated from the samples using TRIzol reagent (Invitrogen, USA), adhering to the manufacturer's protocol. Following treatment with RNase‐free DNase, first‐strand complementary DNA (cDNA) was synthesized employing the HiScript III RT SuperMix for qPCR kit (Vazyme, China). qRT‐PCR was conducted utilizing the SYBR Premix Ex Taq™ RT‐PCR kit (Takara, Japan), in accordance with the manufacturer's instructions. PCR analysis was performed on the LightCycler 480 Real‐Time PCR System (Roche, Switzerland). The expression levels of the target genes were normalized against the wheat *TaActin* gene. The specific primers for each gene are detailed in Table S1.

### Mutagenesis and identification of the non‐necrotic mutants

To validate the mutated gene, an EMS mutant library was constructed using the *necl* mutant. Approximately 8000 seeds were treated with 0.5% EMS solution for 10 h after a seven‐hour pre‐soak in deionized water. Post‐treatment, seeds were rinsed for eight hours and air‐dried. The treated M_1_ seeds were sown in the field in Zhao xian, Hebei province, China. From the M_1_ generation, about 3500 spikes were collected, and 5500 M_2_ plants were screened for non‐necrotic mutants. M_3_ seeds from non‐necrotic M_2_ plants were planted to confirm homozygosity. The coding sequence of *TaCNL* from these mutants was amplified and sequenced to identify mutation sites. Primer details are provided in Table S1.

### Wheat transformation

The full‐length coding sequence of *TaCNL* ^*necl*^ was cloned into the *pBG110* vector to generate the *ProUbi*::*TaCNL* ^*necl*^ construct. Then, the construct was introduced into the common wheat cultivar Fielder through *Agrobacterium*‐mediated transformation, utilising the strain EHA105. Positive transgenic plants were identified using the primer *TaCNL‐Select‐F/R*. The details of the primers are provided in Table S1.

### Subcellular localization

Transient gene expression assays in *N*. *benthamiana* were conducted using *Agrobacterium‐mediated* infiltration, following the established protocol (Yuan *et al*., 2021). The constructs *p35S*::*TaCNL* ^*necl*^ *‐GFP* and *p35S*::*TaCNL* ^*Z39*^ *‐GFP* were individually co‐expressed with the red fluorescent protein *p35S*::*mCherry* and the plasma membrane marker *p35S*::*PIP2‐mCherry* in four‐week‐old *N*. *benthamiana* leaves through *Agrobacterium tumefaciens*‐mediated infiltration (strain GV3101). The plasmolysis assays were performed by incubating the samples in 20% mannitol for 25 min at room temperature. Fluorescence signals were evaluated and captured 36 h post‐injection using a laser confocal scanning microscope (Zeiss LSM980), adhering to the manufacturer's guidelines.

### Bulked Segregant RNA sequencing (BSR‐Seq) analysis

Leaf samples were procured from F_2:3_ families of J411 × *necl* exhibiting homozygous green and necrotic phenotypes in the field. Thirty homozygous green (Bulk‐Green) and homozygous necrotic (Bulk‐Necrotic) lines, each represented by equal‐sized leaf segments from individual plants, were pooled separately. BSR‐Seq was conducted following a pipeline described by Xie *et al*. (2020). In brief, total RNA extraction adhered to the protocol of the TRIzol reagent (Invitrogen). BSR‐Seq was performed using the BGISEQ‐500 platform at the Beijing Genomics Institute (BGI), yielding 109.76 million reads for the Bulk‐Green and 119.46 million reads for the Bulk‐Necrotic. Trimmomatic v0.32 was utilized for the removal of low‐quality reads (Bolger *et al*., 2014), and high‐quality reads were aligned to the SY Mattis v1.0 using the STARv2.5.1b software (Langmead and Salzberg, 2012). SNP variants were identified from unique and confident read alignments employing the ‘HaplotypeCaller’ module of the Genome Analysis Toolkit (GATK) v3.6, with stringent criteria: Fisher's Exact Test (FET) *P*‐value <1e‐8 and allele frequency difference (AFD) > 0.6 (McKenna *et al*., 2010). Only SNPs were used to discern differences between Bulk‐Green and Bulk‐Necrotic. Reads mapped to high‐confidence genes annotated from SY Mattis Annotation PGSBv2.1 in each sample were quantified using RSEM (v1.2.8) with default parameters (Li and Dewey, 2011). Differential expression analysis employed the Bioconductor package ‘DESeq2’, considering genes with an absolute log2 (fold change) ≥ 2 and adjusted *P*‐value ≤0.05 as Differentially Expressed Genes (DEGs) (Wang *et al*., 2010). Gene Ontology (GO) analyses were conducted using the TopGO package. GO terms with a *Q* value ≤0.05 were considered statistically significant. GO enrichment visualization is generated using online tools (https://www.bioinformatics.com.cn/plot_multi_gopathway_enrichment_bubbleplot_016).

### Yeast two‐hybrid assay

The full‐length coding sequence of *TaCNL* ^*necl*^ and *TaCNL* ^*Z39*^ was cloned into the *pGBKT7* vector, respectively. The bait construct *pGBKT7‐TaCNL* ^*necl*^ was transformed into the yeast strain Y2HGold for screening against a yeast cDNA library derived from leaves of Z39. The library screening, plasmid isolation and sequencing were performed by ZOON‐BIO BIOTECHNOLOGY. For 1:1 Y2H assays, the coding sequences of *TaPAL‐6A* and *TaPAL‐6A* ^*AS*^ were separately inserted into the prey vector *pGADT7*. Different combinations of the constructs were co‐transformed into the yeast strain Y2HGold. Yeast cells were first selected on synthetic dropout (SD) medium lacking Leu and Trp (SD‐Leu/‐Trp) and then transferred to SD‐Trp/‐Leu/‐His selective medium with 5 mM 3‐amino‐1,2,4‐triazole (3‐AT) for protein interaction analysis. The primer sequences are listed in Table S1.

### Split luciferase complementation assay

The coding sequences of *TaCNL* and *TaPAL‐6A* ^*AS*^ were cloned into the *pCAMBIA1300‐Cluc* and *pCAMBIA1300‐Nluc* vectors, respectively. Different vector combinations were co‐transfected into *N*. *benthamiana* leaf epidermal cells using *Agrobacterium*‐mediated infiltration. After incubating the injected leaves for 48 h, they were sprayed with 1 mM luciferin (Promega, E1605), and the signal was captured using a CCD imaging apparatus (Berthold, LB985). The primer sequences are listed in Table S1.

### Co‐immunoprecipitation

The coding sequence of *TaCNL* was individually inserted into the *pCAMBIA1300‐221‐MYC* vector, while the coding sequence of *TaPAL‐6A* ^*AS*^ was cloned into the *pCAMBIA1300‐221‐Flag* vector, respectively. These vectors were separately transformed into *A*. *tumefaciens* strain GV3101 and co‐expressed in *N*. *benthamiana* leaves for transient expression with indicated combinations. Total proteins were extracted using extraction buffer, and the lysates were incubated with anti‐MYC magnetic beads (P2118, BEYOTIME) for 0.5 h at 4 °C with rotation. The obtained immunoprecipitated samples were washed more than 5 times with a buffer (150 mM NaCl, 50 mM Tris–HCl (pH 7.4), 1 mM EDTA, 10% (v/v) glycerol, 0.5% (v/v) Triton X‐100, and 1× protease inhibitor cocktail), and then boiled at 65 °C for 5 min in 50 μL 1x SDS‐loading buffer. The samples were then separated by 10% SDS‐PAGE and detected by anti‐Flag (Abmart, M20008) and anti‐MYC (Abmart, M20002) antibodies, respectively. The protein signals were detected using the eECL Western Blot Kit (Abcam, ab65623) and analysed using the Tanon‐4500 gel imaging system according to the manufacturer's instructions. The primer sequences are listed in Table S1.

### Assay of phenylalanine ammonia‐lyase (PAL) activity

The activity of PAL was determined using a UV spectrophotometric method, following the protocol provided in the Phenylalanine Ammonia‐Lyase Activity Assay Kit (BC0210‐50 T, Solarbio). Briefly, tissue samples (0.1 g) were homogenized in 1 mL of extraction buffer and centrifuged at 10000 × *g* for 10 min at 4 °C. The supernatant was collected for subsequent analysis. The reaction mixture (1.0 mL) consisted of 20 μL of sample, 780 μL of Reagent A and 200 μL of Reagent B, and was incubated at 30 °C for 30 min. After the addition of 40 μL of Reagent C, the mixture was allowed to stand for 10 min at room temperature. The absorbance at 290 nm was measured using a UV spectrophotometer. PAL activity was calculated based on the change in absorbance (ΔA) using the following formula: Phenylalanine ammonia‐lyase activities (U g^−1^ FW) = 17.3 × ΔA/W, where W represents the tissue weight (fresh weight, g). For each experimental condition, three leaves were sampled, and the experiment was conducted with three independent biological replicates.

### Barley stripe mosaic virus (BSMV)‐mediated gene silencing

BSMV‐mediated gene silencing in the *necl* mutant was conducted following a previously established protocol (Yuan *et al*., 2021). Specifically, an antisense fragment of *TaPAL‐6A* was cloned into the *pCaBS‐γbLIC* vector to generate the BSMV:*TaPAL* construct. The constructs were individually transformed into the *A*. *tumefaciens* strain EHA105. The resulting agrobacterial cultures were resuspended in infiltration buffer to an optical density (OD600) of 0.5, and the bacterial suspensions were mixed in a 1:1:1 ratio for infiltration into *N*. *benthamiana* leaves. The *pCaBS‐γb* empty vector (BSMV:*γ*) was employed as the negative control. Twelve days post‐injection, leaf sap from *N*. *benthamiana* was collected and utilized to inoculate the ten‐day‐old wheat plants. Following a 12–16 day incubation period, newly developed leaves exhibiting viral symptoms were harvested for subsequent *Bgt* inoculation. For each experimental condition, a minimum of three wheat leaves were selected for analysis, and the experiment was conducted with three independent biological replicates. The expression levels of *TaPAL* were also determined by qRT‐PCR. The primer sequences are listed in Table S1.

### Statistical analysis

All data were analysed using GraphPad Prism 9.0 software (San Diego, CA, USA). Comparisons between groups were evaluated using Student's *t*‐tests or one‐way ANOVA, Tukey's post hoc test (*p* < 0.05). *, ** and *** indicate a statistically significant difference at *P* < 0.05, *P* < 0.01, and *P* < 0.001 levels, respectively.

## Author contributions

H.‐Q. L. and Y.S. planned and designed the project. S.T., Y.S., T.D., S.Z., J.N. and Z.Z. performed the experiments and data analysis. H.L. constructed the *necl* mutant. Q.‐H. S. provided scientific support and advice. Y.S. wrote the manuscript. H.‐Q. L., S.T. and S.Z. revised the manuscript. All authors read and approved the contents of this paper.

## Conflict of interests

The authors declare no competing interests.

## Supporting information


**Figure S1** The *necl* mutant reduces production potential and enhances powdery mildew resistance. (a) Phenotypes of Z39 and the *necl* mutant at the seedling stage in the field. Red arrows indicate the necrosis syndrome on the basal leaves and blade tips. Scale bar, 10 cm. (b) Agricultural trait comparison of Z39 and the *necl* mutant. Data represent the means ± SD (*n* = 25). Student's *t*‐test was used for statistical significance analysis. *** indicates a statistically significant difference at *P* < 0.001 level. ns, no significant difference. (c) The *necl* mutant displayed enhanced powdery mildew resistance. Two‐week‐old Z39 and the *necl* mutant plants were inoculated with the *Bgt* isolate E09. A representative picture was photographed at 7 days post‐inoculation. Scale bar, 8 cm.
**Figure S2** Genetic analysis of the *necl* mutant. Whole plant architecture (top panel), spike (middle panel) and flag leaf (bottom panel) of Z39, the *necl* mutant, and the resulting F_1_ plants are shown.
**Figure S3** Candidate gene associated with the *necl* phenotype is located on chromosome 2B. (a) Manhattan plot for the *necl* phenotype identified by association analysis. The red line indicates the ‐log_10_(*P*‐values) = 5. (b) Local Manhattan plot (0–70 Mb) of chromosome 2B is shown.
**Figure S4** Sequence alignment of the genes in the candidate region cloned from China Spring, the *necl* mutant and SY mattis. Partial sequences of three candidate genes (*TraesCS2B02G058900*, *TraesCS2B02G059000* and *TraesCS2B02G059100*) cloned from China Spring (CS), the *necl* mutant and SY mattis were compared using DNAMAN v5.0.
**Figure S5** Dotplot alignment of the candidate region from SY mattis v1.0 (horizontal) and Chinese Spring v1.0 (vertical). Black dots signify alignments between SY mattis v1.0 and the forward strand of the IWGSC RefSeq v1.0 genome, while red dots denote alignments with the reverse strand.
**Figure S6** 3D structure predictions of TaCNL^Z39^ and TaCNL^*necl*^. The predicted 3D protein structures of TaCNL^Z39^ and TaCNL^*necl*^ were obtained from the AlphaFold Protein Structure Database (https://alphafold.ebi.ac.uk/) and Missense3D (http://missense3d.bc.ic.ac.uk/missense3d/). Green to purple represents the N terminus to C terminus, and red represents the mutation site (Lys421Glu).
**Figure S7** TaCNL^Z39^ interacts with TaPAL‐6A^AS^. (a) Yeast two‐hybrid assays showing the interactions between TaCNL^Z39^ and the active site region (TaPAL‐6A^AS^, residues 107–400) of TaPAL‐6A. (b) The interactions of TaCNL^Z39^ and TaPAL‐6A^AS^ detected by split luciferase complementation assays. (c) Co‐immunoprecipitation assay demonstrating the interaction between TaCNL^Z39^ and TaPAL‐6A^AS^ in *N*. *benthamiana* leaves. MYC beads were employed to immunoprecipitate the TaPAL‐6A^AS^‐Flag protein, and gel blots were probed using anti‐MYC or anti‐Flag antibodies. MYC‐GFP protein was used as the negative control. IP, immunoprecipitation.
**Figure S8** Enrichment bubble map of GO terms for differentially expressed genes between the Bulk‐Green and Bulk‐Necrotic groups. The enrichment score represents the degree to which a specific GO term is overrepresented in a set of differentially expressed genes compared to a background gene set. GO enrichment analysis displayed the first 20 GO terms related to biological process with the most significant enrichment selected by Enrichment Score. Genes related to immune responses are highlighted by black arrows. The size of the dot represents the number of genes, the colour represents the *P*‐value. GO, gene ontology.
**Figure S9** The *TaCNL‐b* allele displays no enhanced powdery mildew resistance. (a) Partial sequence alignment of *TaCNL* ^*Z39*^, *TaCNL* ^*necl*^ and *TaCNL‐b* is shown. The red box indicates the SNP variations. (b) Two‐week‐old Z39, the *necl* mutant, and six wheat accessions with the *TaCNL‐b* allele were inoculated with *Bgt* isolate E09, and representative leaves were photographed at 7 days post‐inoculation. Scale bar, 1 cm. (c) Phenotypes of *Nicotiana benthamiana* leaves injected with vectors encoding HA‐labelled fusion proteins, including TaCNL^Z39^, TaCNL^*necl*^ and TaCNL‐b, are shown (left panel) at 48 h post‐injection. Cell death in *N*. *benthamiana* leaves was observed by trypan blue staining (middle panel). Protein accumulation is shown in the right panel (target bands are highlighted by red arrows). The *BAX* gene was used as the positive control, and the empty vector (EV) was used as the negative control.
**Figure S10** Gene collinearity in the *TaCNL* region among multiple published wheat reference genomes. The microcollinearity among 12 wheat reference genomes (including Chinese Spring, Zang1817, and 10+ genome references) is shown. Genes and lines are grouped by homologous relationships, which are divided into four types: RBH (Reciprocal Best Hit), SBH (Single‐side Best Hit), singleton, and 1‐to‐many (all putative homologous genes). All homologous lines are grouped into three groups by score: 0–50, 50–70 and 70–100. *TaCNL* is marked by the red triangle.
**Figure S11** Phylogenetic tree analysis of *TaCNL*. Homologues of *TaCNL* in published reference genomes of diploid (AA, BB, DD), tetraploid (AABB) and hexaploid (AABBDD) wheat were used to construct a phylogenetic tree using MEGA X software. A total of 112 homologous genes were divided into six groups. The numbers on each branch represent the percentage of gene replication, and different coloured dots represent different ploidy wheat.
**Figure S12** GO enrichment of differentially expressed genes in Bulk‐Necrotic relative to Bulk‐Green groups. GO terms related to biological processes were primarily analysed based on up‐regulated and down‐regulated genes, represented by orange and green bars, respectively. The enrichment score represents the degree to which a specific GO term is overrepresented in a set of differentially expressed genes compared to a background gene set. Genes related to immune responses and photosynthesis are highlighted by black and blue arrows, respectively. GO, gene ontology.
**Figure S13** Ultrastructure of chloroplasts in Z39 and the *necl* mutant. (a) Chloroplast morphology in a single cell of Z39. (b) Ultrastructure of a single chloroplast in Z39. (c) Chloroplast morphology in a single cell of the *necl* mutant. (d) Ultrastructure of a single chloroplast in the *necl* mutant.

**Table S1** Primer sequences used in the study.
**Table S2** Annotated genes within the candidate region based on IWGSC Annotation v1.1.
**Table S3** Annotated genes within the candidate region based on SY Mattis Annotation PGSBV2.1.
**Table S4** Differentially expressed genes of Bulk‐Necrotic group relative to Bulk‐Green group.
**Table S5** Conduct Gene Ontology (GO) enrichment analysis on differentially expressed genes in the Bulk‐Necrotic group compared to Bulk‐Green group.
**Table S6** Phenylalanine catabolic process was up‐regulated in Bulk‐Necrotic group.
**Table S7** Downstream pathway genes associated with PAL are up‐regulated in the *necl* mutant.
**Table S8** Distribution of *TaCNL* in 413 common wheat accessions.
**Table S9** Genetic diversity of *TaCNL*.

## Data Availability

The raw data and sequencing data have been deposited in the National Genomics Data Center (NGDC) database under project CRA022480. The nucleotide sequences of *TaCNL* ^*Z39*^, *TaCNL* ^*necl*^ and *TaCNL‐b* alleles have been uploaded in GenBank with accession numbers PV017274, PV017275 and PV017276, respectively.
