# Supplementary material for: A semi‐dominant NLR allele regulates growth and disease resistance in wheat
Source: Plant Biotechnol J. 2025 Jul 17;23(11):4694–708. doi: 10.1111/pbi.70244 (PMC12576435; doi:10.1111/pbi.70244)
Supplement: Supplementary file 1 — Figure S1 The necl mutant reduces production potential and enhances powdery mildew resistance. (a) Phenotypes of Z39 and the necl mutant at the seedling stage in the field. Red arrows indicate the necrosis syndrome on the basal leaves and blade tips. Scale bar, 10 cm. (b) Agricultural trait comparison of Z39 and the necl mutant. Data represent the means ± SD (n = 25). Student's t‐test was used for statistical significance analysis. *** indicates a statistically significant difference at P < 0.001 level. ns, no significant difference. (c) The necl mutant displayed enhanced powdery mildew resistance. Two‐week‐old Z39 and the necl mutant plants were inoculated with the Bgt isolate E09. A representative picture was photographed at 7 days post‐inoculation. Scale bar, 8 cm. Figure S2 Genetic analysis of the necl mutant. Whole plant architecture (top panel), spike (middle panel) and flag leaf (bottom panel) of Z39, the necl mutant, and the resulting F1 plants are shown. Figure S3 Candidate gene associated with the necl phenotype is located on chromosome 2B. (a) Manhattan plot for the necl phenotype identified by association analysis. The red line indicates the ‐log10(P‐values) = 5. (b) Local Manhattan plot (0–70 Mb) of chromosome 2B is shown. Figure S4 Sequence alignment of the genes in the candidate region cloned from China Spring, the necl mutant and SY mattis. Partial sequences of three candidate genes (TraesCS2B02G058900, TraesCS2B02G059000 and TraesCS2B02G059100) cloned from China Spring (CS), the necl mutant and SY mattis were compared using DNAMAN v5.0. Figure S5 Dotplot alignment of the candidate region from SY mattis v1.0 (horizontal) and Chinese Spring v1.0 (vertical). Black dots signify alignments between SY mattis v1.0 and the forward strand of the IWGSC RefSeq v1.0 genome, while red dots denote alignments with the reverse strand. Figure S6 3D structure predictions of TaCNLZ39 and TaCNL necl . The predicted 3D protein structures of TaCNLZ39 and TaCNL necl were [file PBI-23-4694-s002.pdf]

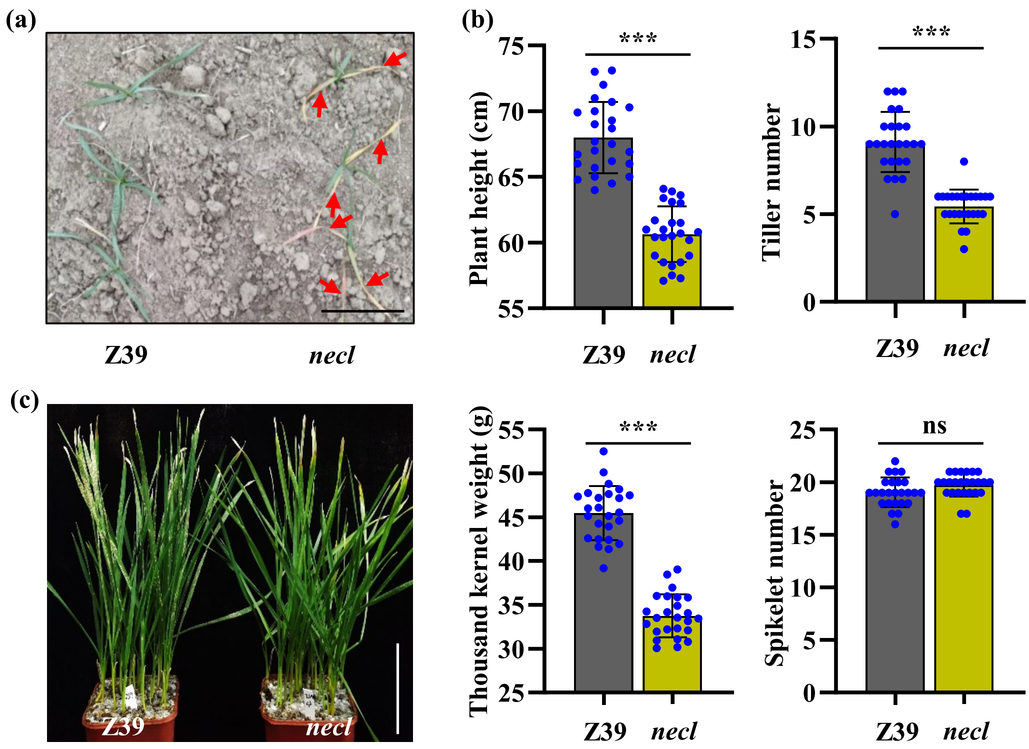

Figure S1 The *necl* mutant reduces production potential and enhances powdery mildew resistance. (a) Phenotypes of Z39 and the *necl* mutant at the seedling stage in the field. Red arrows indicate the necrosis syndrome on the basal leaves and blade tips. Scale bar, 10 cm. (b) Agricultural trait comparison of Z39 and the *necl* mutant. Data represent the means ± SD (n = 25). Student's t-test was used for statistical significance analysis. *** indicates a statistically significant difference at $P < 0.001$ level. ns, no significant difference. (c) The *necl* mutant displayed enhanced powdery mildew resistance. Two-week-old Z39 and the *necl* mutant plants were inoculated with the *Bgt* isolate E09. A representative picture was photographed at 7 days post-inoculation. Scale bar, 8 cm.

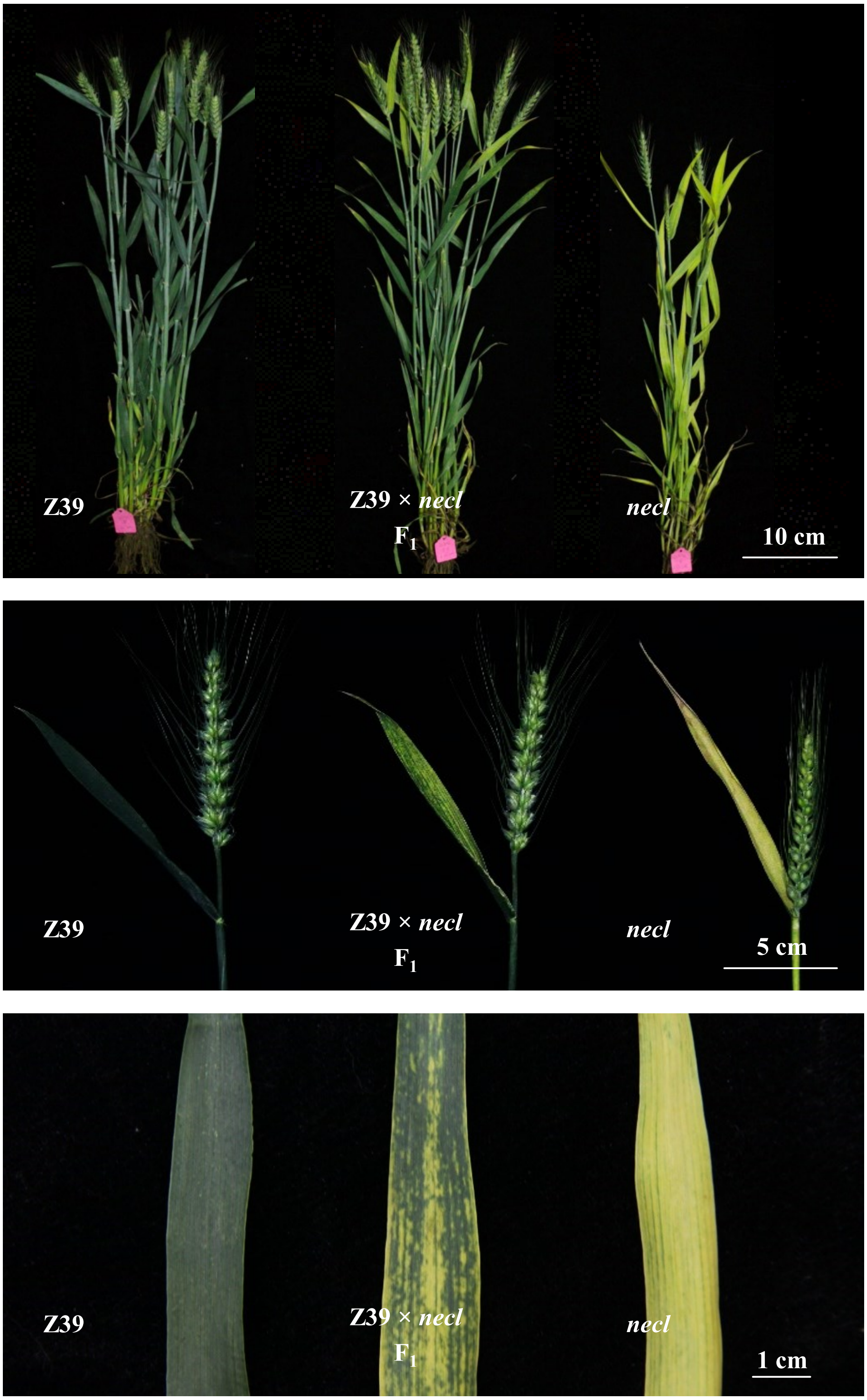

Figure S2 Genetic analysis of the *necl* mutant. Whole plant architecture (top panel), spike (middle panel) and flag leaf (bottom panel) of Z39, the *necl* mutant, and the resulting F_1 plants are shown.

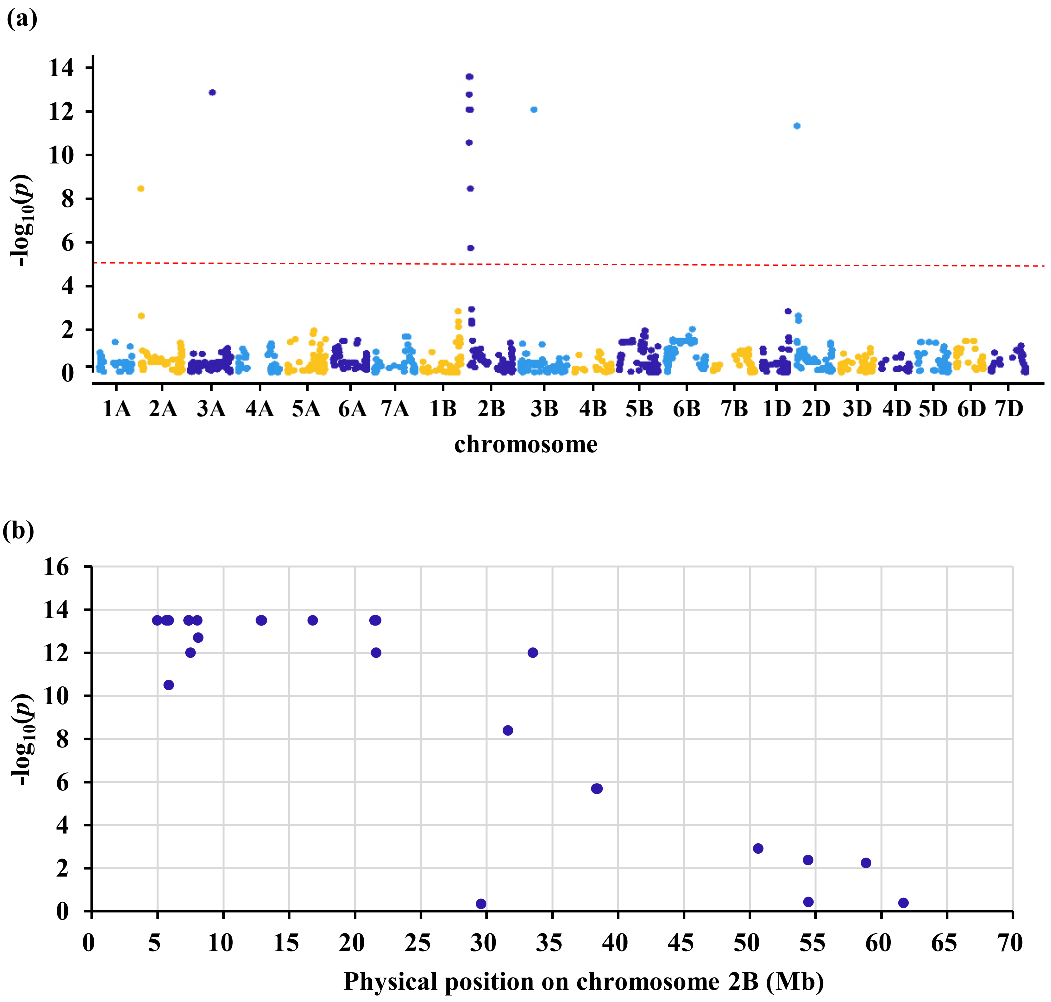

Figure S3 The candidate gene associated with the *necl* phenotype is located on chromosome 2B. (a) Manhattan plot for the *necl* phenotype identified by association analysis. The red line indicates the $-\log_{10}$(*P*-values) = 5. (b) Local Manhattan plot (0–70 Mb) of chromosome 2B is shown.

TraesCS2B02G058900

```
CS        TGGATGTATAAATTGCTAGATAGTACATTCTCTATTTTCAGAAGTATTGGACCTATGGAACCTTTTGAGGGA
necl      TTTATGTATAAAGTGCTGGATAAAGCATTCTCGGTTATCAAATGTGTTGAGCCTATGGAACCGTTTGAGGGA
SY mattis TTTATGTATAAAGTGCTGGATAAAGCATTCTCGGTTATCAAATGTGTTGAGCCTATGGAACCGTTTGAGGGA
          t  atgtataaa tgct gata   cattctc  tt tca a gt ttg  cctatggaacc tttgaggga

CS        TTGTGGAGTTTGAAGAACTTGCAAACTCTGAAGCCAGTTTGTGCTAGTGAAGTTTCCGTCACAAAACTAGCA
necl      TTGTGGAGTTTGAAGAATTTGCAATCCCTGAGGTCCGTTCGAGGTAGTGAAGTTTTTGTTGCAAAACTAGCA
SY mattis TTGTGGAGTTTGAAGAATTTGCAATCCCTGAGGTCCGTTCGAGGTAGTGAAGTTTTTGTTGCAAAACTAGCA
          ttgtggagtttgaagaa ttgcaa c ctga g c gtt g g tagtgaagttt  gt  caaaactagca

CS        AATTTATCTCAGTTAAGGACCCTTAGCATTATTGGGGTAAGGAGCATCCACTGTGCACAACTCTGTGCATCG
necl      AATTTATCTCAGCTGAGGACCCTTAGCATTATAGGGGTAAGGAACATCCACTGTGCACAACTGTGTGACTCT
SY mattis AATTTATCTCAGCTGAGGACCCTTAGCATTATAGGGGTAAGGAACATCCACTGTGCACAACTGTGTGACTCT
          aatttatctcag t aggacccttagcattat ggggtaagga catccactgtgcacaact tgtg  tc
```

TraesCS2B02G059000

```
CS        GTGTTTTCTAAGCGG....ATATAGTGTGTCAAGTGTCAACAAGGCATCACTGGACAGATACTAATCTTTAA
necl      GTGTTTTCTAAGCGGATATATATAGTGTGTCAAGTGTCAACAAGGCATCATTGGACAGATACTAATCTTTAT
SY mattis GTGTTTTCTAAGCGGATATATATAGTGTGTCAAGTGTCAACAAGGCATCATTGGACAGATACTAATCTTTAT
          gtgttttctaagcgg    atatagtgtgtcaagtgtcaacaaggcatca tggacagatactaatcttta

CS        GTGTATGCCTTATGGTTGAATATACCCGTCCAGTTTTGAGTAGATCTAGAAATTAGACCCACAAGAACTCCA
necl      GTGTATGCCTTATGGTTGAATATACATGTTGAGTTTTGAGTAGATCTAGAAATTTGACCCACAGGAAGTCCA
SY mattis GTGTATGCCTTATGGTTGAATATACATGTTGAGTTTTGAGTAGATCTAGAAATTTGACCCACAGGAAGTCCA
          gtgtatgccttatggttgaatatac  gt  agttttgagtagatctagaaatt gacccaca gaa tcca

CS        CAGCAAGGGGGCCTAAAACCCTCTCCATGTCATACCATAAACCATCGTTCGACTCCCTAGAAGGCCAGCTTA
necl      CAATAAGA.GGCCTAAAACCCTCTCCATGTCACACCATAAACCC....TCGACTCCCAAGAAGGCCAGCTTA
SY mattis CAATAAGA.GGCCTAAAACCCTCTCCATGTCACACCATAAACCC....TCGACTCCCAAGAAGGCCAGCTTA
          ca  aag  ggcctaaaaccctctccatgtca accataaacc     tcgactccc agaaggccagctta
```

TraesCS2B02G059100

```
CS        TGTGTAGCTGCATCGTCCAGATGGCCACAAAGTTTAGAGGAATAAATATTTGAGAAACGGCCGAA.......
necl      TGTGTAGCTGCATCGTCCAGATGGCCAGAAGGTTTAGAGGAATAAATATTTGAGAAACGGCCGAAATCTCCA
SY mattis TGTGTAGCTGCATCGTCCAGATGGCCAGAAGGTTTAGAGGAATAAATATTTGAGAAACGGCCGAAATCTCCA
          tgtgtagctgcatcgtccagatggcca aa gtttagaggaataaatatttgagaaacggccgaa

CS        ......ATCTTATGCAAACTCATGAAACAGAAAATCTAAAAGAACTCATGAAACTGAAAGACTAATGGTGAA
necl      TGCGCTATCTGATGCAAACTCATGAAACTGAAAATCTAAAAGAACTCATGGAACTGAAAGACTAATGGTG.A
SY mattis TGCGCTATCTGATGCAAACTCATGAAACTGAAAATCTAAAAGAACTCATGGAACTGAAAGACTAATGGTG.A
                atct atgcaaactcatgaaac gaaaatctaaaagaactcatg aactgaaagactaatggtg a

CS        TGTATTCTTGACATTGTGGAAAATAATATGTCACTGGAATCCTATATATCTAAGAAGGTCAT
necl      TGTATTCTTGACATTGTGAGTAAAAAAATGTCGCTGGAATTCTATATACCTAAAAAGGTCAT
SY mattis TGTATTCTTGACATTGTGAGTAAAAAAATGTCGCTGGAATTCTATATACCTAAAAAGGTCAT
          tgtattcttgacattgtg   aa aa atgtc ctggaat ctatata ctaa aaggtcat
```

Figure S4 Sequence alignment of the genes in the candidate region cloned from China Spring, the *necl* mutant and SY mattis. Partial sequences of three candidate genes (*TraesCS2B02G058900*, *TraesCS2B02G059000* and *TraesCS2B02G059100*) cloned from China Spring (CS), the *necl* mutant and SY mattis were compared using DNAMAN v5.0.

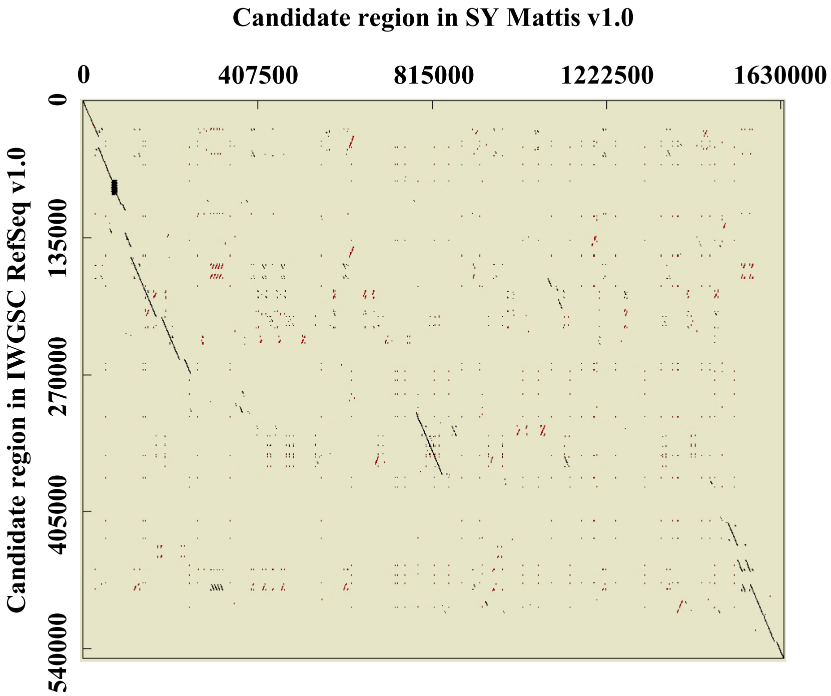

Figure S5 Dotplot alignment of the candidate region from SY mattis v1.0 (horizontal) and Chinese Spring v1.0 (vertical). Black dots signify alignments between SY mattis v1.0 and the forward strand of the IWGSC RefSeq v1.0 genome, while red dots denote alignments with the reverse strand.

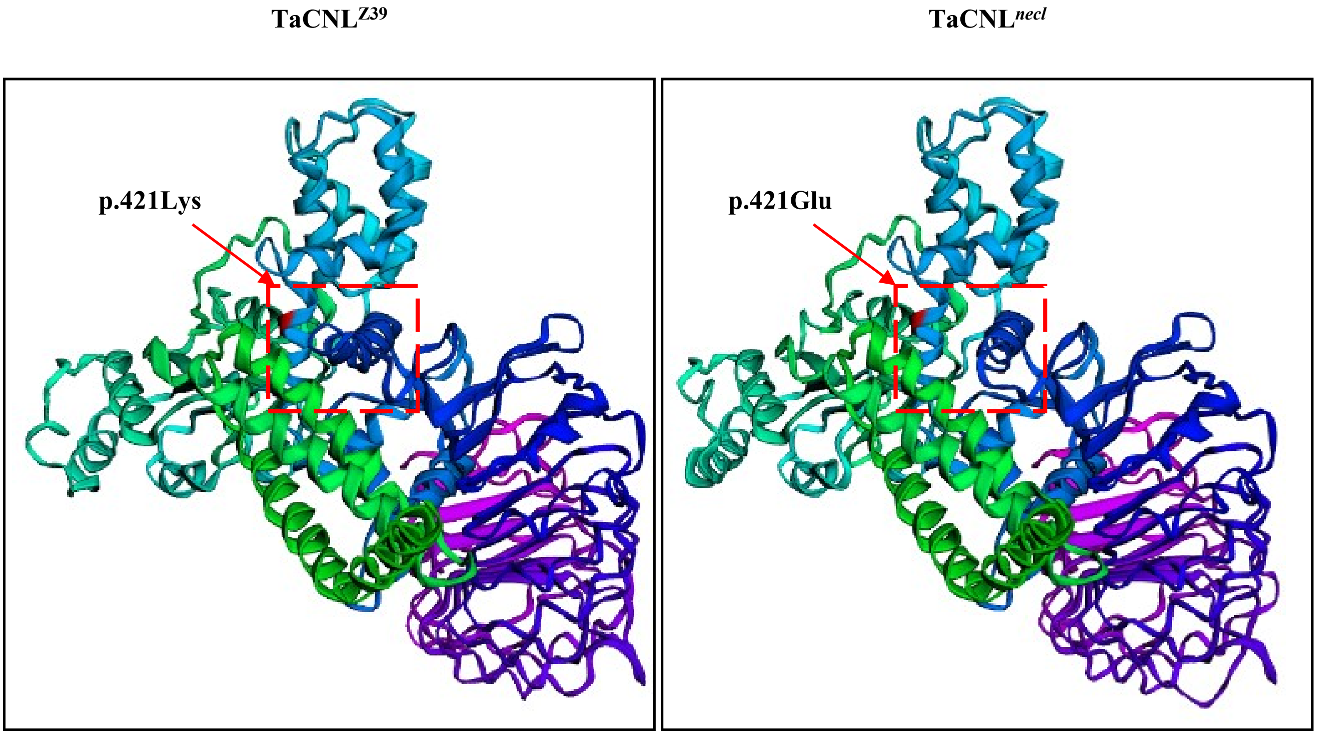

Figure S6 3D structure predictions of TaCNL^{Z39} and TaCNL^{necl}. The predicted 3D protein structures of TaCNL^{Z39} and TaCNL^{necl} were obtained from the AlphaFold Protein Structure Database (https://alphafold.ebi.ac.uk/) and Missense3D (http://missense3d.bc.ic.ac.uk/missense3d/). Green to purple represents the N terminus to C terminus, and red represents the mutation site (Lys421Glu).

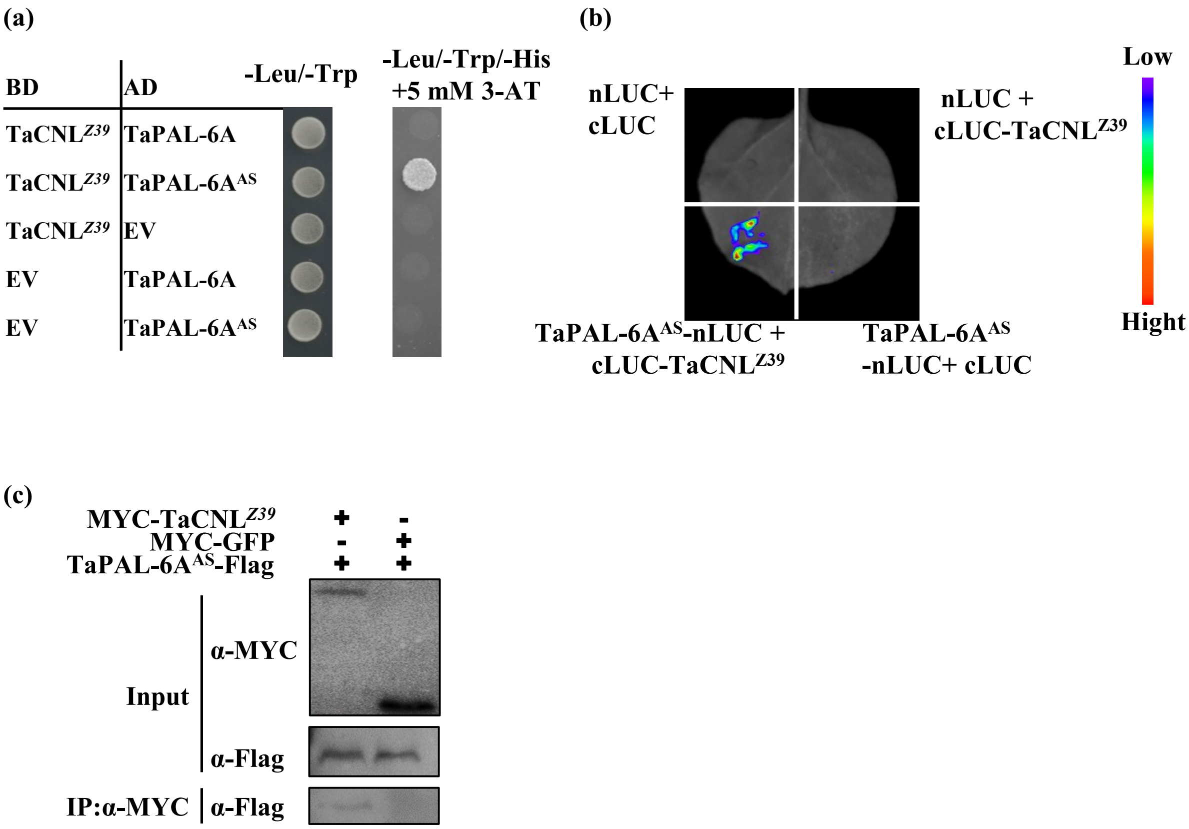

Figure S7 TaCNL^{Z39} interacts with TaPAL-6A^{AS}. (a) Yeast two-hybrid assays showing the interactions between TaCNL^{Z39} and the active site region (TaPAL-6A^{AS}, residues 107－400) of TaPAL-6A. (b) The interactions of TaCNL^{Z39} and TaPAL-6A^{AS} detected by split luciferase complementation assays. (c) Co-immunoprecipitation assay demonstrating the interaction between TaCNL^{Z39} and TaPAL-6A^{AS} in *N. benthamiana* leaves. MYC beads were employed to immunoprecipitate the TaPAL-6A^{AS}-Flag protein, and gel blots were probed using anti-MYC or anti-Flag antibodies. MYC-GFP protein was used as the negative control. IP, immunoprecipitation.

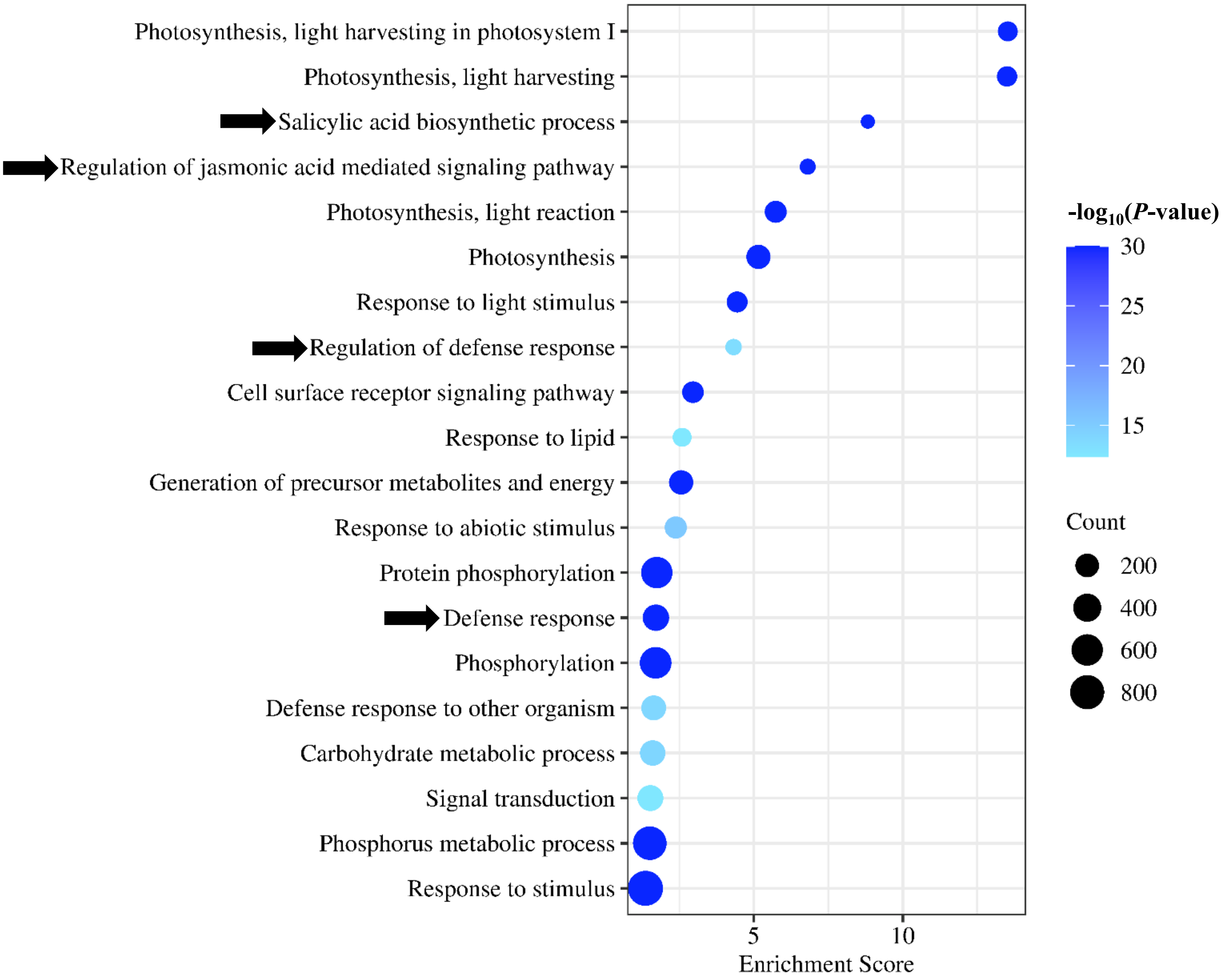

Figure S8 Enrichment bubble map of GO terms for differentially expressed genes between the Bulk-Green and Bulk-Necrotic groups. The enrichment score represents the degree to which a specific GO term is overrepresented in a set of differentially expressed genes compared to a background gene set. GO enrichment analysis displayed the first 20 GO terms related to biological process with the most significant enrichment selected by Enrichment Score. Genes related to immune responses are highlighted by black arrows. The size of the dot represents the number of genes, the colour represents the *P*-value. GO, gene ontology.

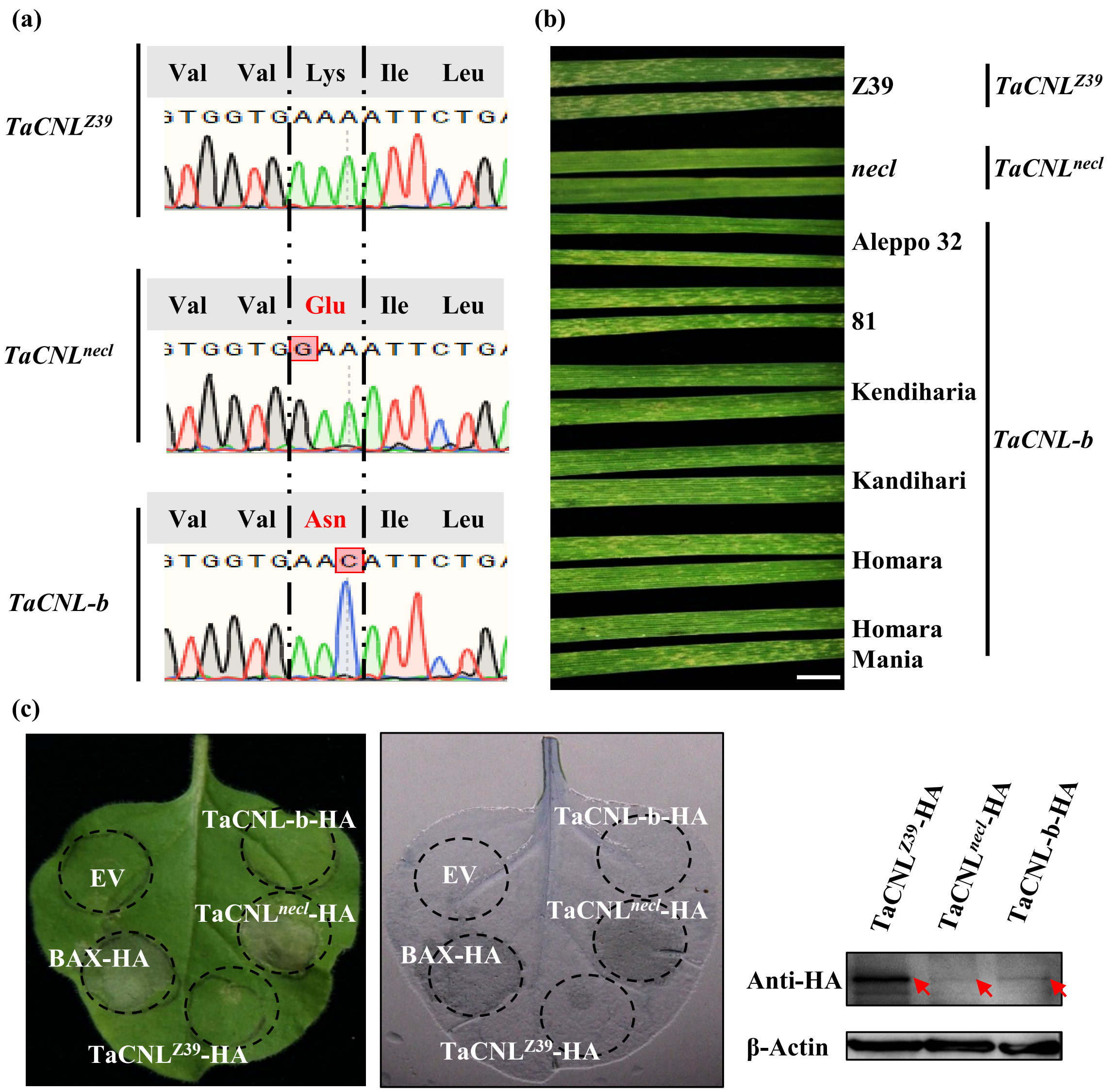

Figure S9 The *TaCNL-b* allele displays no enhanced powdery mildew resistance. (a) Partial sequence alignment of *TaCNL*^{Z39}, *TaCNL*^{necl} and *TaCNL-b* is shown. The red box indicates the SNP variations. (b) Two-week-old Z39, the *necl* mutant, and six wheat accessions with the *TaCNL-b* allele were inoculated with *Bgt* isolate E09, and representative leaves were photographed at 7 days post-inoculation. Scale bar, 1 cm. (c) Phenotypes of *Nicotiana benthamiana* leaves injected with vectors encoding HA-labelled fusion proteins, including TaCNL^{Z39}, TaCNL^{necl} and TaCNL-b, are shown (left panel) at 48 h post-injection. Cell death in *N. benthamiana* leaves was observed by trypan blue staining (middle panel). Protein accumulation is shown in the right panel (target bands are highlighted by red arrows). The *BAX* gene was used as the positive control, and the empty vector (EV) was used as the negative control.

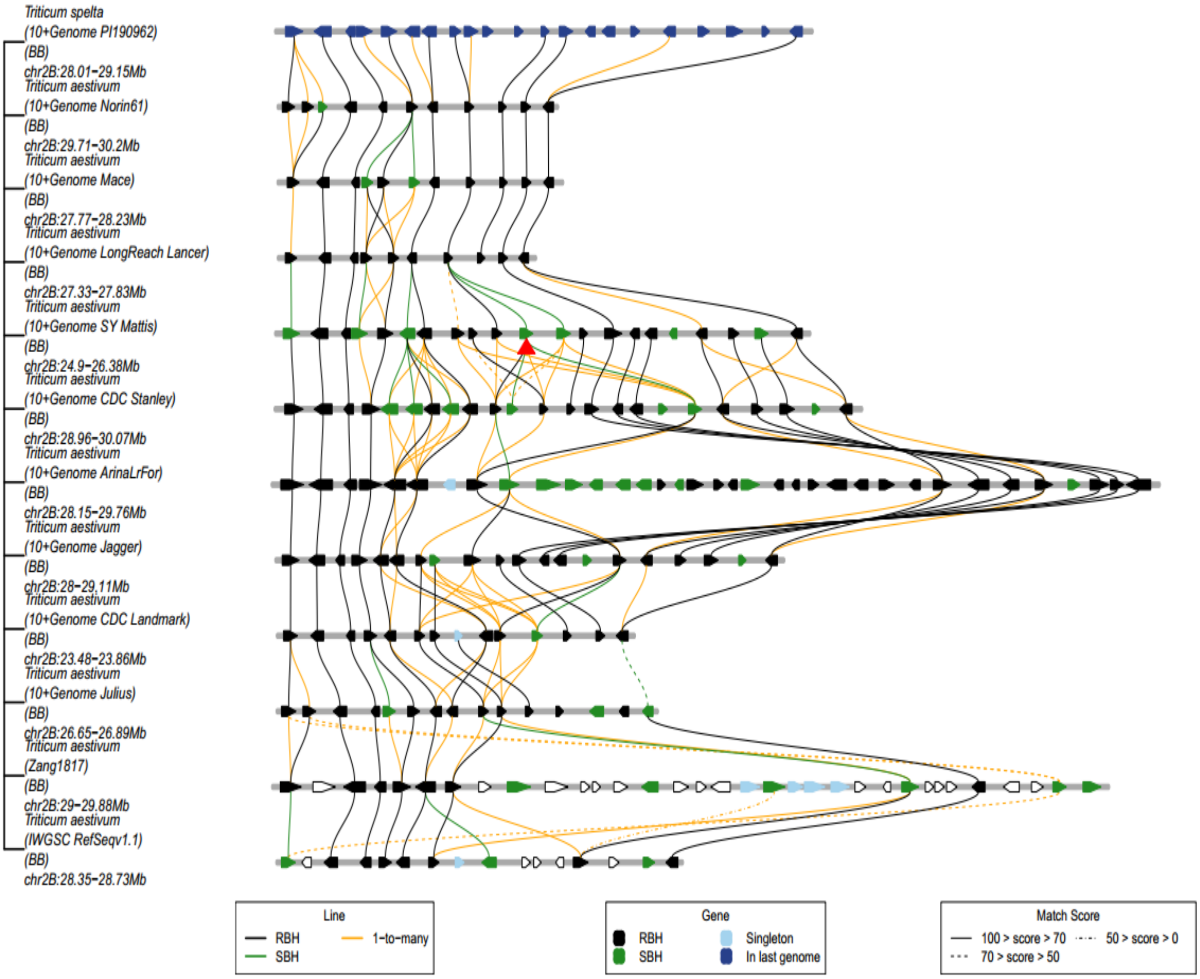

Figure S10 Gene collinearity in the *TaCNL* region among multiple published wheat reference genomes. The microcollinearity among 12 wheat reference genomes (including Chinese Spring, Zang1817, and 10+ genome references) is shown. Genes and lines are grouped by homologous relationships, which are divided into four types: RBH (Reciprocal Best Hit), SBH (Single-side Best Hit), singleton, and 1-to-many (all putative homologous genes). All homologous lines are grouped into three groups by score: 0-50, 50-70 and 70-100. *TaCNL* is marked by the red triangle.

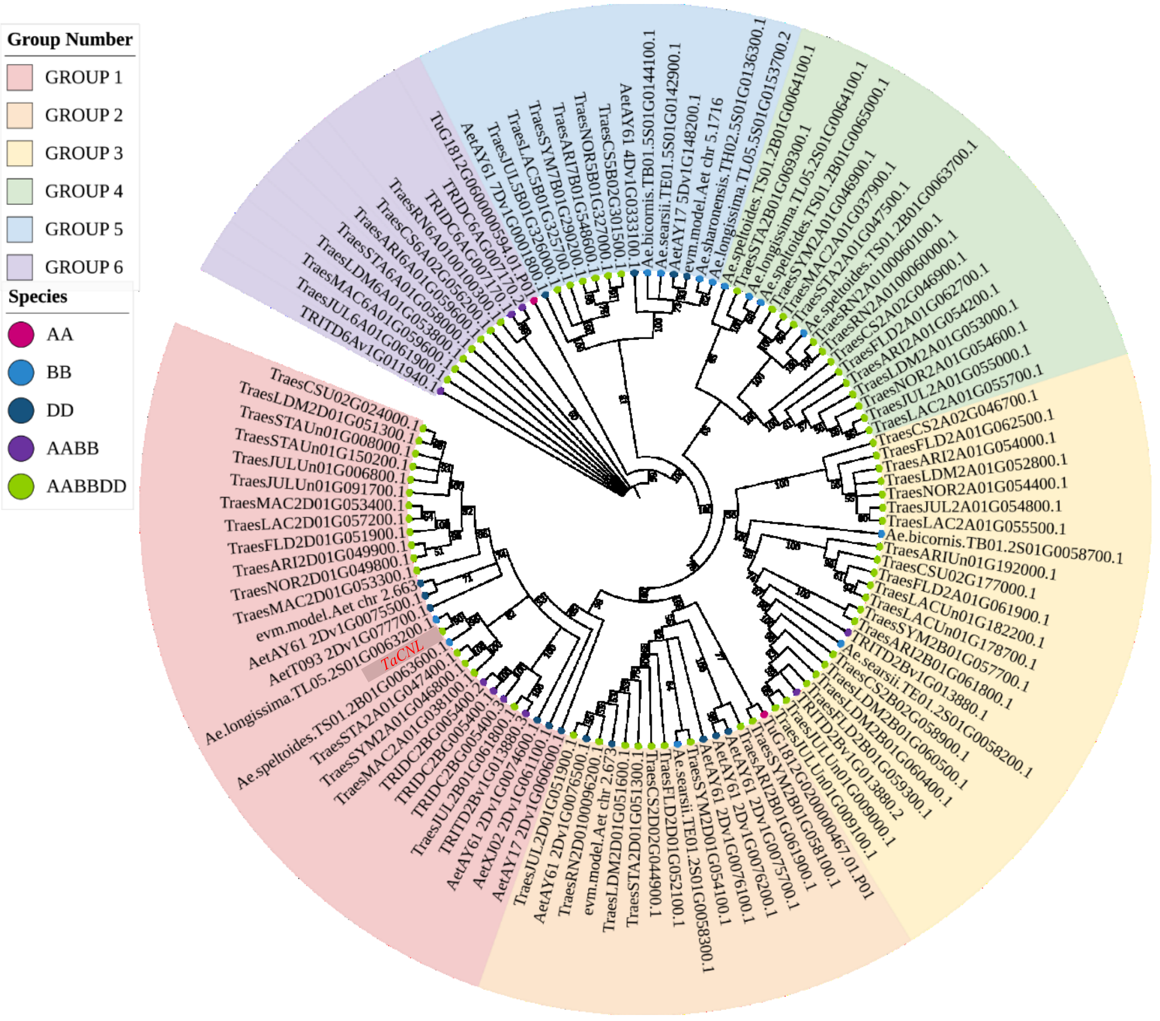

Figure S11 Phylogenetic tree analysis of *TaCNL*. Homologues of *TaCNL* in published reference genomes of diploid (AA, BB, DD), tetraploid (AABB) and hexaploid (AABBDD) wheat were used to construct a phylogenetic tree using MEGA X software. A total of 112 homologous genes were divided into six groups. The numbers on each branch represent the percentage of gene replication, and different coloured dots represent different ploidy wheat.

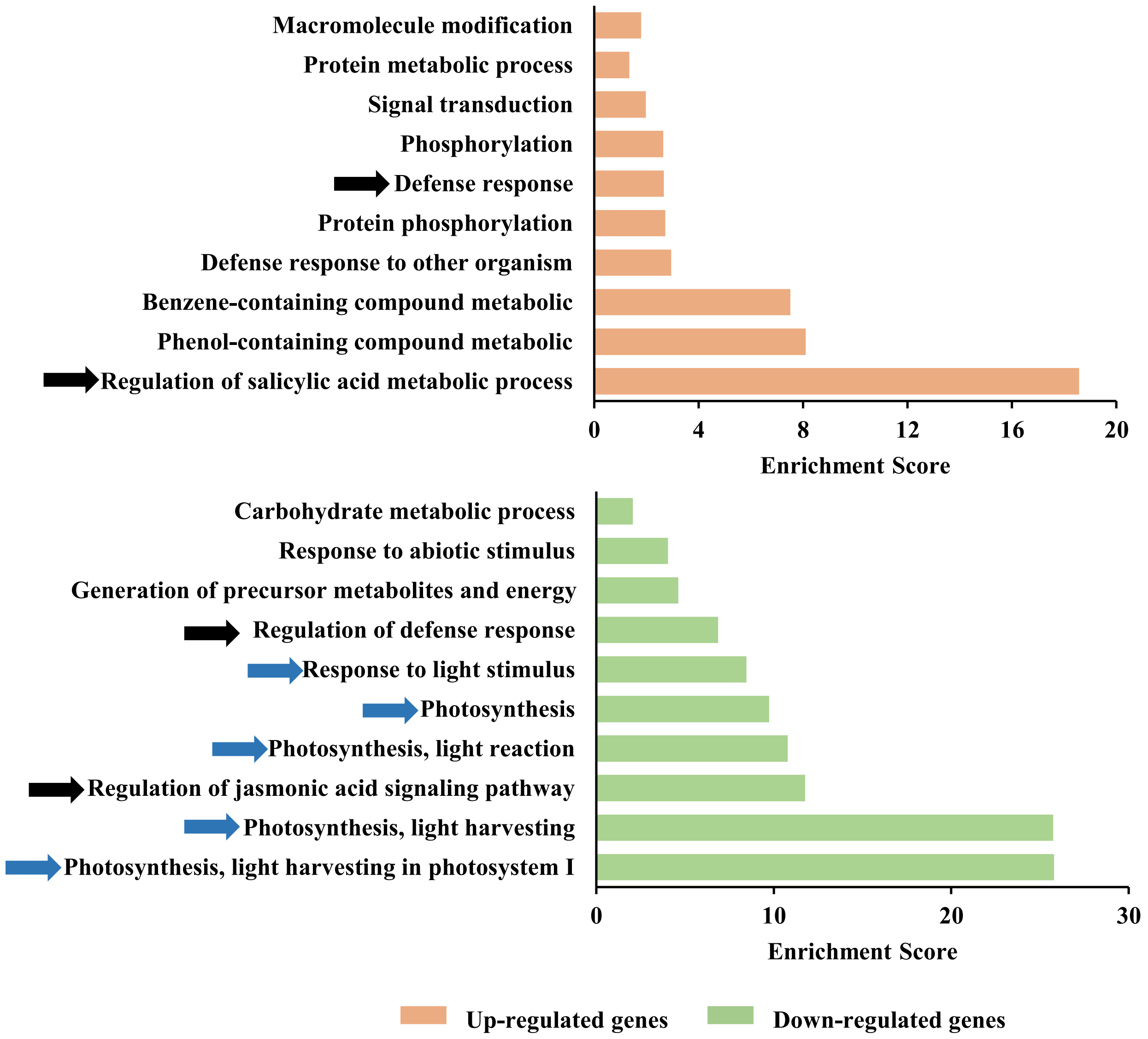

Figure S12 GO enrichment of differentially expressed genes in Bulk-Necrotic relative to Bulk-Green groups. GO terms related to biological processes were primarily analysed based on up-regulated and down-regulated genes, represented by orange and green bars, respectively. The enrichment score represents the degree to which a specific GO term is overrepresented in a set of differentially expressed genes compared to a background gene set. Genes related to immune responses and photosynthesis are highlighted by black and blue arrows, respectively. GO, gene ontology.

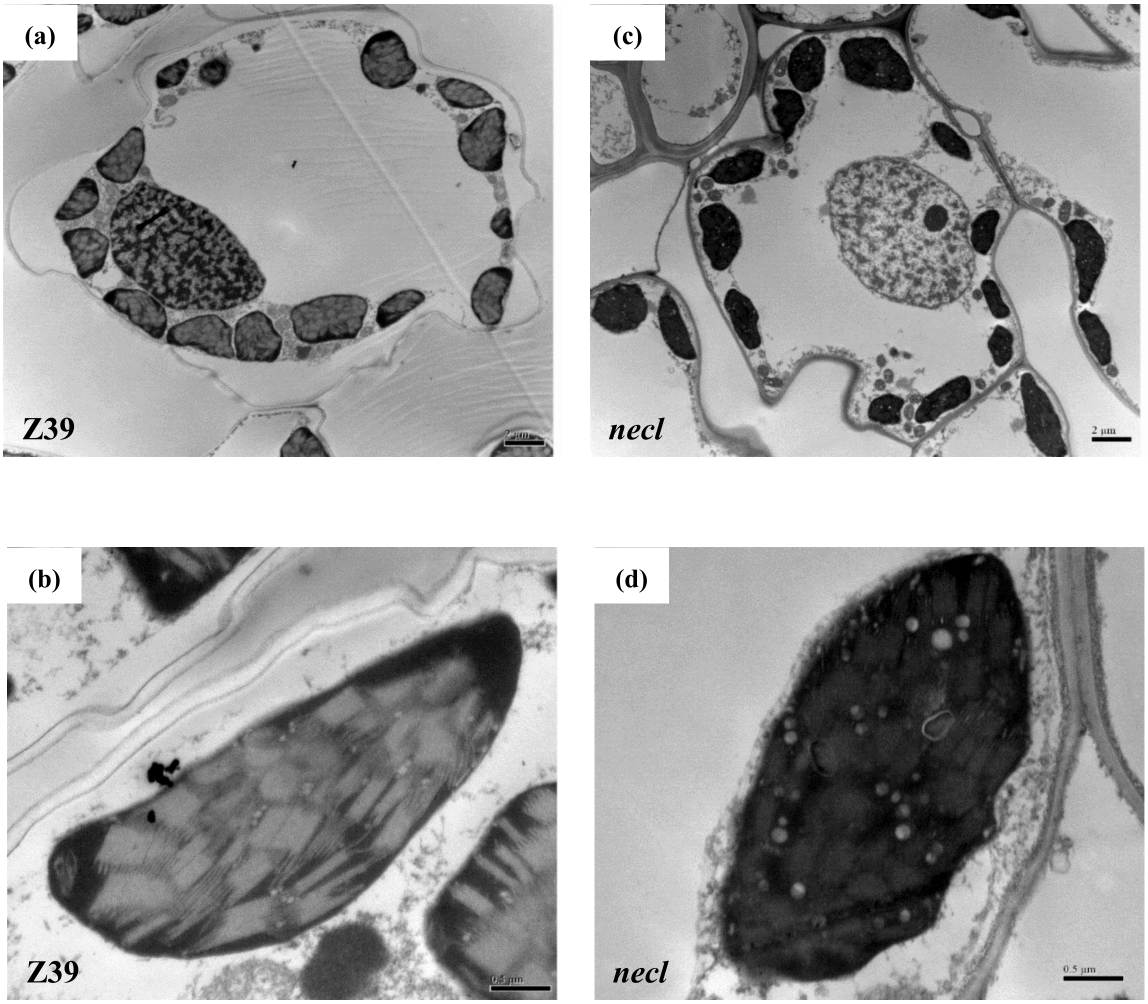

Figure S13 Ultrastructure of chloroplasts in Z39 and the *necl* mutant. (a) Chloroplast morphology in a single cell of Z39. (b) Ultrastructure of a single chloroplast in Z39. (c) Chloroplast morphology in a single cell of the *necl* mutant. (d) Ultrastructure of a single chloroplast in the *necl* mutant.
